# Blood–Brain Barrier and Neurodegenerative Diseases—Modeling with iPSC-Derived Brain Cells

**DOI:** 10.3390/ijms22147710

**Published:** 2021-07-19

**Authors:** Ying-Chieh Wu, Tuuli-Maria Sonninen, Sanni Peltonen, Jari Koistinaho, Šárka Lehtonen

**Affiliations:** 1 Neuroscience Center, University of Helsinki, 00014 Helsinki, Finland; ying.wu@helsinki.fi (Y.-C.W.); tuuli-maria.sonninen@uef.fi (T.-M.S.); sannipel@student.uef.fi (S.P.); jari.koistinaho@helsinki.fi (J.K.); 2 A.I.Virtanen Institute for Molecular Sciences, University of Eastern Finland, 70211 Kuopio, Finland

**Keywords:** induced pluripotent stem cell (iPSC), blood–brain barrier (BBB), neurodegenerative diseases (NDDs)

## Abstract

The blood–brain barrier (BBB) regulates the delivery of oxygen and important nutrients to the brain through active and passive transport and prevents neurotoxins from entering the brain. It also has a clearance function and removes carbon dioxide and toxic metabolites from the central nervous system (CNS). Several drugs are unable to cross the BBB and enter the CNS, adding complexity to drug screens targeting brain disorders. A well-functioning BBB is essential for maintaining healthy brain tissue, and a malfunction of the BBB, linked to its permeability, results in toxins and immune cells entering the CNS. This impairment is associated with a variety of neurological diseases, including Alzheimer’s disease and Parkinson’s disease. Here, we summarize current knowledge about the BBB in neurodegenerative diseases. Furthermore, we focus on recent progress of using human-induced pluripotent stem cell (iPSC)-derived models to study the BBB. We review the potential of novel stem cell-based platforms in modeling the BBB and address advances and key challenges of using stem cell technology in modeling the human BBB. Finally, we highlight future directions in this area.

## 1. Introduction

According to the World Health Organization (WHO), Alzheimer’s disease (AD) and other forms of dementia ranked as the seventh leading cause of death in 2019. The number of people affected by neurodegenerative diseases (NDDs) is estimated to increase due to the elderly population increasing worldwide. However, effective treatments are not yet available because we do not deeply understand the causes of the diseases and therefore do not reach the right targets for therapeutic intervention. NDDs are characterized by the continuing loss of specific populations of neurons resulting from various underlying mechanisms. The most common pathology of NDDs is the abnormal aggregation or inappropriate processing of proteins, such as beta-amyloid (Aβ) in AD and alpha-synuclein (α-synuclein) in Parkinson’s disease (PD) [1]. Proteasome impairment, mitochondria dysfunction, and immune system imbalance have also been revealed as potential mechanisms for NDDs [2]. However, numerous evidence now also link vascular dysfunction to NDDs. Vascular alterations are discovered in most NDDs, particularly in capillaries. These alterations have been shown to cause vasculature dysfunction and contribute to the disease progression of NDDs in recent studies [3,4,5]. As the vessels are three-dimensional (3D) structures composed of several cell types that are hard to imitate by the in vitro model, most of the studies are performed in mouse models. However, mouse models have been proved to inaccurately mimic all human disease phenotypes. Therefore, a human cell-based model is still required in this field. With the development of iPSCs technology, BBB studies have been benefited from it. On the one hand, iPSCs have theoretically unlimited sources of cells and can differentiate into any type of human cells. These properties counteract the limitation in the amount and lifespan of isolated primary human brain vascular cells. Moreover, iPSCs carry the genetic information from the donor, which enables developing a patient-specific NDDs model for mechanism studying and drug screening [6]. Recent studies have used iPSC-derived cells to build more complicated co-culture BBB models such as transwell, spheroid, and organ-on-chip. These co-culture models mimic the structural and functional characteristics of the BBB and provide more insights into the vascular function and cell interaction studies.

In this review, we first describe the basic function of the specific vasculature in the brain, forming the blood–brain barrier (BBB), and how the BBB breaks down in NDDs, including AD, PD, amyotrophic lateral sclerosis (ALS), and Huntington’s disease (HD). Then, we focus on the protocols generating BBB cell types and methods modeling the BBB with iPSC. Furthermore, we summarize results on BBB alterations in NDDs using patient-specific iPSC models from recent studies. Finally, we discuss future directions in the field and the opportunities to build an effective model to investigate the pathology of NDDs and find novel treatment targets.

## 2. The Blood–Brain Barrier

The BBB comprises several cell types: endothelial cells (ECs), astrocytes, and pericytes [7]. These cells work together to form a selectively permeable membrane. The BBB prevents the brain from circulating pathogens and toxins and helps with maintaining brain homeostasis [8,9]. ECs are a major part of establishing the barrier function, while other cell types regulate the BBB function through different signaling pathways. Below, we will discuss the role of each cell type in the BBB and how they support the BBB functions.

### 2.1. Endothelial Cells

ECs establish the walls of blood vessels. They can form larger vessels by gathering dozens of cells [9]. Brain ECs closely connect and hold each other by the tight junctions (TJ) proteins such as claudin, occludin, and ZO-1. TJs act as a barrier, limiting the paracellular transportation of molecules [10,11]. In addition, vesicle trafficking is particularly low in brain ECs that restrict the transcytosis through the cells. Transcytosis is suppressed by forkhead box protein F2 (Foxf2) signaling from pericytes or major facilitator superfamily domain-containing 2a (Mfsd2a) produced by brain ECs themselves [12,13,14]. These features allow the BBB to limit the entrance of blood-borne solutes. Most molecules cross the BBB through active transport except for water, oxygen, and small lipid-soluble substances that can pass the BBB by diffusion [7]. To deliver essential nutrients into the brain, brain ECs have a higher expression level of nutrient transporters such as glucose transporters (GLUT) and L-amino acids transporter (LATs) than peripheral ECs [15,16,17]. In addition, brain ECs express an exceedingly low level of leukocyte adhesion molecules (LAMs) in health conditions. ECs in other tissues recruit leukocytes from the blood to tissue to eliminate pathogens, while brain ECs with low-level LAM expression limit the infiltration of peripheral immune cells into the brain to prevent neuroinflammation [18,19].

Furthermore, brain ECs also develop systems to remove waste from the brain. For example, astrocytes produce lactate via glycolysis as an energy source for neurons and supporting long-term memory formation in neurons. To avoid excessive lactate accumulation in the brain that becomes toxic for cells, brain ECs export lactate by proton-linked monocarboxylate transporter 1 (MCT1) [20,21]. MCT1 was also found to have a higher expression level in brain ECs than peripheral ECs [22]. In pathological conditions, studies also reveal that brain ECs are able to transport amyloid from the brain to blood through low-density lipoprotein receptor-related protein-1 (LRP1) and receptor for advanced glycation and products (RAGE) [23,24]. These results indicate the importance of waste and toxin removal via the BBB transporter system.

Overall, these unique properties of the brain ECs ensure that the brain gains sufficient nutrients and stays in a stable environment. However, these beneficial features cause problems for treating brain disorders as well. Due to the limited types of molecules that can pass the BBB, most drugs targeting the brain cannot pass the BBB, which leaves us with big challenges.

### 2.2. Astrocytes

Astrocytes extend their processes forming glia limitans that separate the neuronal cells and blood vessels. Glia limitans ensheath alongside blood vessels, limiting unwanted molecules entering into the brain [9,25]. Astrocytes can regulate BBB function through astrocyte-derived factors or physical contact with ECs and pericytes. Potassium channel Kir4.1 and aquaporin-4 (AQP-4) are enriched in astrocyte endfeet and are crucial for maintaining water and potassium homeostasis. The absence of Kir4.1 and AQP-4 causes an imbalance of water and ions, thus affecting the BBB and leading to abnormal capillary permeability [26,27,28]. Additionally, astrocytes are reported to have dual effects on BBB regulation [29]. Astrocytes can release cytokines and chemokines, promoting or disrupting the barrier integrity that depends on the signal they receive from neurons or other cell types. For example, matrix metalloproteinases (MMPs) and nitric oxide (NO) produced by astrocytes break down BBB via disrupting TJ proteins and induce the inflammatory process [29,30,31,32,33]. On the contrary, angiopoietin-1 (ANG-1) and glial cell line-derived neurotrophic factor (GDNF) secreted from astrocytes promote barrier function through the cytoskeleton reorganization pathway [34,35,36]. Additionally, astrocytes are able to regulate the expression level of intercellular adhesion molecule-1 (ICAM-1) and vascular cell adhesion molecule-1 (VCAM-1) on ECs, which relate to the leukocyte attachment and transition [37]. In vitro co-culturing of ECs and astrocytes also improves endothelial integrity comparing to EC monoculture [38]. One study also shows that the leakage of plasma protein from blood to the CNS by the conditioned ablation of astrocytes in adult mice cannot be compensated by other cell types in the BBB [39]. These results indicate that astrocytes are necessary for BBB maintenance.

### 2.3. Pericytes

Pericytes are cells embedded in the basement membrane and cover the abluminal side of the EC layer [40]. When forming new vessels, ECs recruit pericytes by secreting transforming growth factor-beta (TGF-β) and platelet-derived growth factor-BB (PDGF-BB) [41]. The depletion of pericytes in both the developmental and adult stages of mice increases the vascular permeability, indicating endothelial barrier dysfunction [42,43]. These results reveal the importance of pericytes in vascular development and stabilization. In addition, pericyte loss in any stage of development and adulthood may cause vascular dysregulation and lead to BBB breakdown.

Pericytes also play an important role in modulating BBB function. Studies show that pericytes can release the signaling factors or regulate the gene expression level of other cell types in the BBB. For instance, in vitro co-culture of ECs and pericytes elevate TJ proteins expression level in ECs compared to EC monoculture. The expression level of leukocyte adhesion-related genes in ECs is upregulated in pericyte-deficient mice, which may lead to immune cell infiltration into the brain [42]. In addition, pericytes are able to control EC sprouting and cell cycle progression through vascular endothelial growth factor (VEGF) signaling and contractility [44]. Moreover, pericyte deficiency affects the polarization of astrocyte endfeet and results in BBB dysfunction [43].

In specific circumstances, pericytes also show the potency to differentiate into other lineages. Sakuma et al. found that platelet-derived growth factor receptor (PDGFR) positive pericytes express stem cell markers Nestin and SRY (sex-determining region Y)-box 2 (Sox2) after ischemic stroke. Then, these cells turn into microglia-like cells [45]. In Nakagomi et al., these stem cell marker positive pericytes were isolated and cultured in different condition mediums. The group discovered that these pericytes could differentiate into vascular and neural lineages depending on the guidance factors [46]. In Özen et al., pericytes were also found to acquire microglial phenotypes after stroke [47]. These studies suggest that pericytes act as stem cell reservoirs after tissue injury to compensate for the loss of other cell types.

It is noteworthy that pericytes are able to regulate immune response as well. Pericytes express receptors for pathogen/damage-associated molecular patterns and can propagate inflammatory responses while receiving these signals [48,49]. Studies show that inflammatory cytokines secreted by pericytes mediate the polarization of microglia toward promoting or inhibiting inflammatory phenotype [50]. Additionally, pericyte stimulation by lipopolysaccharide (LPS) is found to produce pro-inflammatory factors such as NO, interleukin (IL)-10, and IL-17, which may participate in the neuroinflammation process [51].

## 3. Blood–Brain Barrier Dysfunction in Neurodegenerative Diseases

### 3.1. Alzheimer’s Disease

AD is a progressive neurologic disorder known as the most common cause of dementia. Memory impairment and decline in cognitive function are typical characteristics in AD patients [52]. These symptoms arise from the continuous neuron and glia cell loss in the brain, especially in the hippocampus and entorhinal cortex [53]. Amyloid plaques, neurofibrillary tangles, and cerebral amyloid angiopathy (CAA) are hallmarks of AD [52,54]. More than 90% of AD cases are sporadic, and apolipoprotein E (ApoE) is considered as the main genetic risk factor for sporadic AD. There are three major variants of ApoE in humans (ApoE2, ApoE3, and ApoE4). ApoE4 allele is strongly associated with AD, while ApoE3 is the most common variant and considered neutral (usually as the control in ApoE-related research) [55,56]. Mutations in amyloid precursor protein (APP), presenilin-1 (PS1), and presenilin-2 (PS2) are the cause of familial AD, in which symptoms appear much earlier than sporadic cases [57]. According to the amyloid cascade hypothesis, Aβ deposition is believed to be the initial event of AD [54]. However, increasing evidence shows that cerebrovascular dysfunction may contribute to AD progression as well. Below, we will discuss how the BBB acts in the aged and diseased brain and how these changes emerge during NDD, with a focus on AD.

#### 3.1.1. Accumulation of Aβ and Phosphorylated Tau in Cerebrovasculature

The accumulation of Aβ around blood vessels is usually described as CAA, which is a common age-related small vessel disease. CAA is not only found in AD patients but also highly correlated with dementia. The exact origin of vascular amyloid is not clear, but studies suggest that it is mainly produced by neurons and deposited in the vasculature due to an imbalance of production and clearance [58]. Depositions of Aβ thicken vessel walls and narrow the luminal space, from which emerges cerebral microbleeds and hemorrhages [59,60]. In addition, lesions in subcortical white matter can be found in the post-mortem brains of patients with CAA, which is associated with cognitive function decline [60].

Accumulation of Aβ in pericytes was also observed in AD patients and APP Swedish mutation mice. In addition, studies revealed that pericyte loss is correlated with increased Aβ depositions in AD human and mice brains [61,62]. In vitro studies have shown increased apoptosis activity and reduced pericyte proliferation after exposure to fibrillary Aβ 1-42, leading to overall pericyte survival reduction [63]. These results may indicate the mechanism of how Aβ depositions cause pericyte loss in vivo. In addition, studies reported that vascular amyloid displaces astrocyte endfeet in the AD mouse model. Displaced astrocyte endfeet no longer lean on the arteriole layer, which disrupts the integrity of glia limitans [64]. These results suggest that Aβ accumulation in vasculature may induce pericyte death and alter astrocyte morphology, leading to BBB dysfunction.

Tau is another major pathology hallmark of AD. In the AD brain, tau is abnormally phosphorylated and aggregates to form the neurofibrillary tangles (NFT) [65]. Several studies have pointed out that tau also contributes to the BBB dysfunction in AD. In AD mice (APP overexpression and Swedish mutation), depositions of oligomeric tau are found in cerebrovasculature [66]. Tau depositions induced morphology changes in blood vessels, including increasing atrophic string capillaries and irregularities on the capillary surface [67,68,69]. Reduced blood vessels diameter and thickened vessel walls were also found in AD mice with NFT [68,70]. In addition, cerebral blood flow (CBF) was negatively correlated with tau expression level. CBF reduction is a typical phenotype in AD patients and relates to cognitive decline [71]. In addition, tau is associated with TJ proteins reduction in the cortical region of AD brains [72]. According to these studies, it is clear that tau/NFT induces blood vessels morphology changes and vascular dysfunction. However, how tau/NFT interacts with BBB cell types remains unclear. More studies need to be done to understand the underlying mechanisms.

#### 3.1.2. Endothelial Cell Dysfunction

Impairment of BBB function in AD patients results from several reasons, and one of the major ones is the degenerated endothelium. The MMP-9 protein in brain ECs is present in many brain disorders, including AD [73]. The activation of MMP-9 causes TJ proteins disruption and extracellular matrix (ECM) degradation, which leads to endothelial barrier dysfunction [74]. Furthermore, increased expression and subsequent activation of MMP proteins are reported to activate the immune response and contribute to neuroinflammation [75]. A decrease in TJ proteins also relates to Aβ accumulation. One study shows that the TJ expression level is negatively correlated with the insoluble Aβ level in the cortex of human post-mortem tissues [76]. In addition, VE-cadherin, a component of endothelial adherens junctions that is important for maintaining vascular integrity, was also reported to decrease in AD patients and mouse brains [77].

Glucose transport is also affected in AD patients. The expression level of GLUT1 is reduced in brain capillaries in both human AD patients and mouse AD models [78,79]. Since glucose is the main energy nutrient for the brain, impaired glucose transport leads to neurodegeneration and brain atrophy [79]. Since a reduction in glucose uptake in the brain may occur in the early stage of AD progression, it may become an effective biomarker for AD diagnosis [80]. P-glycoprotein 1 and LRP-1 are transporters known to remove Aβ from the brain across the BBB. Both P-glycoprotein 1 and LRP-1 are significantly reduced in AD brain ECs, which decreases Aβ clearance from the brain and accelerates AD progression. In comparison, another transporter related to Aβ delivery, RAGE, is upregulated in the AD brain. Since RAGE delivers Aβ in the opposite direction, from blood to the brain, the upregulation of RAGE also increases Aβ deposition in the AD brain [81,82,83,84].

Additionally, studies show that the transcription profiling of ECs in the AD brain is altered. The homeobox gene, mesenchyme homeobox 2 (MEOX2), which relates to vascular differentiation, has a lower expression level in AD patients. Reductions in the density of brain capillaries, impaired Aβ efflux, and loss of the angiogenic response to hypoxia are found in mice with deleted MEOX2, suggesting the importance of MEOX2 in regulating endothelial functions [85].

In Lau et al., the single-nucleus transcriptomic analysis also revealed alterations of brain ECs in genes involved in angiogenesis and immune response in AD patients. The group identified seven subclusters of ECs via subcluster analysis and found that three of them contributed to the transcriptomic changes in AD. Genes associated with angiogenesis (Claudin 5 (CLDN5), ETS-related gene (ERG), Fms Related Receptor Tyrosine Kinase 1 (FLT1), and von Willebrand factor (VWF)) were enriched in the three subpopulations. Further pathway analysis revealed that these angiogenic ECs are regulated by several genes (Interferon Gamma (IFNG), Interferon Regulatory Factor 7 (IRF7), and T-cell receptor (TCR)) related to the inflammatory response. These results suggest that a pro-inflammatory response regulates angiogenic ECs induction, and ECs may contribute to altered angiogenesis and immune response in AD pathogenesis [86].

#### 3.1.3. Astrocyte Dysfunction

For a long time, neurons have been considered as the only cell type able to produce Aβ. However, astrocytes are found to produce a detectable amount of Aβ in the PS1 and APP Swedish mutation mouse brain [87,88,89]. Since astrocytes ensheath the vasculature structure, they may also contribute to the CAA pathology and disrupt BBB integrity in AD.

Astrogliosis is a common feature in NDDs, including the activation and proliferation of astrocytes. Increased glial fibrillary acidic protein (GFAP), vimentin, and synemin expression levels can be considered as markers of astrogliosis [90,91]. Reactive astrocytes can be further classified into ‘A1′ or ‘A2′ astrocytes according to their functions. A1 astrocytes secrete toxic factors that kill surrounding cells, especially neurons, while A2 astrocytes appear to play a neuroprotective role after brain injury [91,92,93,94]. One study reported that A1 astrocytes are highly abundant during the early stages of CAA pathology in AD mice, which further secrete neurotoxins to trigger neuron death and neuroinflammation [95].

In addition, astrocyte degeneration is observed in the AD brain. The atrophic astrocytes have been found in both AD human post-mortem tissues and the mouse brain. These atrophic astrocytes show a reduction in complexity, volume, and GFAP expression level [96,97,98]. Since astrocytes are important for supporting BBB structure, the degeneration of astrocytes in vasculature may disrupt BBB integrity. In addition, the expression level of AQP-4 is increased in astrocytes in AD patients. AQP-4 is important for water homeostasis and relates to capillary permeability [99,100]. Alteration of AQP-4 expression may result in vascular dysfunction in AD patients.

#### 3.1.4. Pericyte Dysfunction

Reduced pericyte coverage on brain capillaries is found in AD patients, and the pericyte coverage rate is negatively correlated with the permeability of the BBB [101,102]. In addition, pericyte loss is found to be more severe in AD patients who carry ApoE4 alleles than those with ApoE3 alleles [102]. The accumulation of cyclophilin A (CypA) and MMP-9 protein is found in pericytes in the AD brain, likely contributing to TJ proteins downregulation in ECs and possibly disturbing the pericyte-EC adhesion [102,103]. In AD patients harboring ApoE4 alleles, CypA and MMP-9 deposition are found more in pericytes compared to AD patients with ApoE3 alleles [102]. These results suggest that different variants of ApoE might affect pericyte attachment to ECs.

Pericyte uptake of Aβ requires LRP1 and apolipoprotein [61,104]. Silencing the ApoE gene in a mouse model results in impairment of Aβ uptake by these cells. This phenotype can be restored by the treatment of astrocyte-derived lipidated human ApoE3 but not ApoE4 [61]. This result indicates that people carrying ApoE4 alleles may have defects in Aβ clearance via pericytes and have Aβ accumulation in the vasculature, which may further lead to CAA pathology.

Studies also reveal that pericytes are able to regulate gene expression of ECs. For example, the TJ protein expression level is increased in pericyte–EC co-culture compared to EC monoculture [42,105]. However, the co-culture of ECs with mice pericytes expressing human ApoE4 downregulates the expression level of ECM protein coding genes compared to ECs cultured with ApoE3 pericytes, where ECM proteins are critical for stabilizing ECs [105]. The endothelial barrier integrity is also reduced in co-culture with ApoE4 pericytes as opposed to those cultured with ApoE3 pericytes [105,106]. These results indicate that ApoE isoforms can differentially modulate the endothelial gene expression and further influence the BBB integrity. Studies have also shown that pericytes might be crucial for Aβ accumulation in the vasculature site. Blanchard et al. co-cultured ApoE3 or ApoE4 endothelial, pericytes, and astrocytes together in different combinations and treated them with Aβ to see the clearance of Aβ by this self-assembled BBB. They found that the BBBs containing ApoE4 pericytes have more Aβ accumulation than those BBBs with ApoE3 pericytes, which suggests that the amyloid phenotype increased with ApoE4 pericytes [107].

As mentioned above, a reduction of CBF is a typical phenotype in AD patients and is related to brain atrophy and cognitive decline. The blood flow even can reach around 50% in some brain regions [108]. Although the pathology is not well-understood, studies suggest that constriction of the capillary pericytes triggered by Aβ deposition may contribute to the phenotype [5,109]. Another possible mechanism has been carried out in the sporadic model. The expression of ApoE4 in mice and lack of murine ApoE leads to BBB breakdown by activating the CypA–nuclear factor-κB (NF-κB)–MM9 pathway in pericytes. Activation of this pathway results in microvascular and CBF reduction, being independent of the Aβ deposition [110,111]. These studies indicate the importance of pericytes in regulating blood flow, which is crucial for AD progression.

These results reveal that pericytes lose their ability to support and regulate ECs when carrying the ApoE4 variant, which contributes to vascular dysfunction. However, the role of pericytes in amyloid pathology, especially in familial AD, remains unclear. On the other hand, these results may also indicate that neurovascular breakdown is crucial for sporadic AD progression due to apolipoprotein involvement in maintaining pericytes and normal barrier functions.

#### 3.1.5. Peripheral Immune Cell Infiltration

The infiltration of peripheral immune cells occurs in many brain disorders such as stroke, trauma, and NDDs. When inflammatory factors or Aβ depositions are released from the brain, the circulating immune cells are attracted and move toward the CNS [112,113,114]. However, the peripheral immune cells do not easily pass through the BBB in physiological conditions. The infiltrating of leukocytes is controlled by the ligand pairing between leukocytes and brain ECs LAMs. Briefly, leukocytes are captured and rolling on ECs mediated by selectins that are expressed on ECs. Then, chemokine release induces the G protein-coupled receptor (GPCR) pathway, which leads to integrins activation on leukocytes. The activated integrins on leukocytes bind to ECs counter-ligands and form firm adhesion with ECs. Finally, leukocytes transmigrate through ECs via platelet endothelial cell adhesion molecule-1 (PECAM-1) signaling [115,116,117]. In healthy conditions, the LAM expression level is extremely low on brain ECs [18,19]. Yet, LAM expression is observed to increase in the AD brain, and the blockade of LAMs on brain ECs reduces leukocyte infiltration [118,119]. Additionally, in pericyte-deficient mice, the LAM expression level was upregulated in brain ECs, which indicates that pericytes might be regulating the LAM expression level [42,43]. The infiltration of immune cells may release reactive oxygen species (ROS) and cytokines contributing to BBB deconstruction. In addition, the cells that migrated through the BBB may activate resident microglia in the brain, resulting in chronic inflammation and neuron degeneration [114].

Taken together, studies have revealed that ECs, astrocytes, and pericytes all have defects in AD brains, indicating the association between BBB dysfunction and AD progression. As the hallmark of AD, Aβ depositions also contribute to BBB breakdown. Accumulation of Aβ in vasculature affects the survival and morphology of BBB cells, thus increasing the vascular permeability. In addition to the Aβ pathology, the degeneration of ECs, astrocytes, and pericytes is observed in AD brains. ECs degeneration leads to a reduction of TJs and dysregulation of transporter activities. Impairments of astrocytes and pericytes result in loss of their ability to support ECs functions and stabilize the vascular structure. All these pathological alterations accumulate and result in BBB disruption. Disrupted BBB increases the permeability to blood-borne immune cells and molecules and further aggravates AD development.

### 3.2. Parkinson’s Disease

Parkinson’s disease (PD) is the second most common NDD after AD. PD is characterized by the loss of dopaminergic neurons (DA) in substantia nigra [107] (SN) pars compacta and the presence of Lewy bodies, which are intracellular inclusions mainly formed by insoluble α-synuclein [120,121]. The main symptoms are motor (tremor, rigidity, and slowness with walking), but several non-motor symptoms are also common. While most cases are sporadic, around 20 disease-associated genes have been identified, including mutations in *LRRK2*, *SNCA*, *PINK1*, *Parkin*, and *GBA*. Despite the fact that several molecular mechanisms have been identified in PD, including α-synuclein pathology, neuroinflammation, mitochondrial dysfunction, and impaired protein degradation, the exact cause of PD is still unknown.

Initially, it was thought that the BBB stays intact in PD, but several studies have now identified its disruption in PD patients. The first clues came from studies that found increased uptake of [(11)C]-verapamil in PD patients, suggesting a decreased P-glycoprotein (P-gp) function [122,123]. More recently, deceased P-gp expression in PD patients was linked to the vitamin D pathway [124]. Further imaging studies have identified increased leakiness in the BBB in basal ganglia regions [125] and cerebral microbleeds, which were more common in PD patients with dementia [126]. Histological analyses have shown capillary leakages and an accumulation of serum proteins in PD patients, as well as evidence of endothelial degeneration (loss of ECs in the basal ganglia, reduced levels of TJ proteins, and alteration of the capillary basement membrane) [127,128]. Increased albumin and IgG cerebrospinal fluid (CSF)/serum ratios have been observed in PD patients [129,130]. Additionally, increased CSF biomarkers of angiogenesis were found in PD patients, and they were associated with gait difficulties, BBB dysfunction, white matter lesions, and cerebral microbleeds [131].

Toxins-induced animal models have been traditionally used to study the disease mechanisms of PD [132]. The most common ones are neurotoxin models, and especially 6-hydroxydopamine (6-OHDA) and 1-methyl-4-phenyl-1,2,3,6-tetrahydropyridine (MPTP) are widely used. When administrated to the brain parenchyma, 6-OHDA produces hydrogen peroxide, superoxide radicals, quinones, and hydroxyl radicals, leading to DA neurons loss. The BBB disruption has been identified in 6-OHDA animal models. 6-OHDA administration had shown to cause BBB leakage, increased P-gp immunoreactivity, and iron accumulation [133,134]. In a more recent study, a 6-OHDA-induced mouse model had a decreased P-gp level associated with the vitamin D receptor pathway [124]. Unlike 6-OHDA, MPTP can be administrated systemically, since it can easily cross the BBB. In the brain, MPTP is metabolized to toxic MPP+ ions by astrocytes. MPP+ is taken up by DA neurons and causes the degeneration of DA neurons [135]. MPTP-treated mice have increased the leakage of albumin into the SN and striatum [136,137]. In addition, the infiltration of immune cells has been detected with this model [136].

The role of PD-related mutations in BBB dysfunction has not been well explored. Studies have indicated that *LRRK2* and *PINK1* mutations have a role in inflammation and regulate monocyte adhesion. In primary human microvascular ECs, PINK1G309D, the loss-of-function mutation associated with early-onset familial PD, increased the expression of VCAM-1 and enhanced the attachment of monocytes to brain ECs [138]. Similarly, the LRRK2 G2019S mutation, which is the most common disease-causing mutation, exacerbated the expression of VCAM-1 and increased the monocyte attachment to ECs [139].

Aggregates of insoluble α-synuclein are typical hallmarks of PD. α-Synuclein is a small 14 kDa protein highly expressed in neurons. In addition to neurons, it is also expressed in low amounts in microglia, astrocytes, and macrophages. In the periphery, α-synuclein is found in red blood cells, platelets, and immune cells. In pathological conditions, α-synuclein can trigger synaptic failure and activate neuroinflammation through microglia and astrocytes. α-Synuclein is also expressed in ECs and can be transported across the BBB in a bi-directional way [140,141]. Higher plasma levels of exosomal α-synuclein have been found in PD patients, suggesting increased efflux to the peripheral blood [142]. Post-mortem studies have associated α-synuclein aggregates with endothelial degeneration and decreased P-gp expression [124,143]. Recent studies have linked α-synuclein-related BBB dysfunction to activated pericytes. In rat brain ECs and pericytes co-culture, monomeric α-synuclein induced the release of inflammatory cytokines from pericytes and increased the permeability of brain ECs [144]. A mouse model overexpressing human α-synuclein showed vascular pathology and BBB disruption, and these changes were accompanied by pericyte activation [145].

### 3.3. Amyotrophic Lateral Sclerosis

Amyotrophic lateral sclerosis (ALS) is an NDD characterized by loss of motor neurons leading to progressive paralysis, muscle atrophy, and finally death, often only a few years from the first symptoms. ALS can be familial (10%), caused by inheritable mutations, or sporadic (90%) with no clear genetic linkage. Mutations in at least 15 different genes, such as *C9orf7*, *SOD1*, *TARDBP*, and *FUS*, have been recognized to underlie familial cases of ALS [146]. It is suggested that the sporadic cases of ALS would arise from a combined effect of environmental and genetic risk factors. Some genes associated with a higher risk for sporadic ALS have been identified, but the identification of environmental factors has not been very successful [147].

In several post-mortem studies, changes related to BBB and blood–spinal cord barrier breakdown in both familial and sporadic ALS were identified. These changes include reduced TJ protein expression, extravasation of red blood cells causing an accumulation of blood-derived proteins, a decreased amount of pericytes in the spinal cord, a detachment of astrocytic endfeet from the endothelium, and enlarged perivascular spaces [148,149,150,151,152,153]. In animal studies, mice with SOD1^G93A^ (dismutase-active) mutation and their offspring with WT mice are the most commonly used in vivo models [151,154,155,156,157]. In addition, rats with SOD1^G93A^ [158,159,160,161,162,163] and mice with other mutations in the *SOD1* gene—SOD1^G37R^ (dismutase-active) and SOD1^G85R^ (dismutase-inactive) [154] have been used in ALS research. These studies showed disruption of the BBB in the early and late stages of the disease [155] and the degeneration of capillary ECs, astrocytes, and motor neurons [155]. In addition, dissociation between endothelium and astrocyte foot processes and swollen astrocyte endfeet affecting the function of the BBB and the blood–spinal cord barrier were detected [150,154,157], as well as neuroinflammation in different regions of the brain and T-cell infiltration in regions with BBB breakdowns [160,162]. Changes in the expression of aquaporin-4 and potassium channel 4.1 in astrocytes indicate a changed ability to maintain water and potassium homeostasis [156,161], reduction of TJs leading to microhemorrhages [154], and accumulation of blood-derived hemoglobin and iron in the blood–spinal cord barrier, leading to a degeneration of motor neurons [159].

The pathology of ALS is not fully understood. However, it has been suggested that the decreased number of TJs and loss of ECs and pericytes during BBB breakdown make it possible for red blood cells and plasma-derived proteins to enter the brain. In the brain, the red blood cells degrade and release neurotoxic hemoglobin and free iron, leading to ROS formation, which is toxic to motor neurons. Additionally, some plasma-derived proteins can initiate non-autonomous motor neuron cell death via microglia [146].

Aggregation of the TDP-43 protein seems to be important in the pathophysiology of the disease. TDP-43 aggregates have been identified in the cytoplasm of motor neurons in the spinal cord, neurons in the hippocampus and frontal cortex, and glial cells in most familial and sporadic ALS (although not in cases with mutations in SOD1) [164,165].

### 3.4. Huntington’s Disease

Huntington’s disease (HD) is caused by a mutation in the *HTT* gene coding for the huntingtin protein. The mutation adds CAG repeats to the gene, causing protein aggregation, leading to neurodegeneration [146]. The repeat length of CAG varies between individuals, where the normal range is 6–26 repeats and will not cause HD. However, people with 40 or more repeats will develop HD with 100% certainty at some stage of life [166].

The mutated form of the huntingtin protein and some of its aggregates are toxic to cells. Oligomers of mutated huntingtin are thought to be more toxic than monomers, larger aggregates, and cleaved proteins [166]. As such, monomers and different-sized aggregates of the huntingtin protein may have distinct effects in cells. The mutated huntingtin proteins can be toxic in several particular ways. They can directly interfere with transcription [167,168], affect vesicular transport [169], disturb the transfer of degraded and misfolded proteins out of the cells [170,171], alter mitochondrial dynamics [172,173], and affect the calcium signaling to initiate apoptosis [174].

Even though huntingtin affects neurons all around the CNS, the first apparent changes are visible in the striatum and globally in the cerebral white matter [175,176]. However, in the late stage of the disease, neuronal loss is already widely spread in the CNS [177]. Mutated huntingtin aggregates are also found in many other cell types: brain ECs, perivascular macrophages, and the vascular basal lamina in patients with HD, suggesting that mutated huntingtin spreads from neurons to other cells [178]. HD also affects the BBB, as the amount of TJ proteins is reduced, and pericyte coverage is lower than in the healthy brain, leading to increased permeability of the BBB [178,179]. In addition, increased microvascular angiogenesis has been observed in HD [178].

Several different mouse lines have been used as in vivo models in studies of BBB dysfunction in HD. The most used mouse line in these studies was R6/2 mice with 150–240 CAG repeats [178,179,180,181,182]. The studies with R6/2 mice have shown increased expression of vascular endothelial growth factor A in astrocytes [179], leading to increased vascular density [178,179,180], impaired BBB permeability [178,181], reduced number of TJs, increased number of vesicles (some containing huntingtin aggregates) [178], decreased vascular reactivity, and decreased pericyte coverage [179]. Other mouse HD models used in studying BBB—Hdh150Q and GFAP-HD/N171-82Q mice—have also shown increased vessel density that was not detected with N171-82Q and GFAP-HD mice [179].

We have reviewed the BBB changes in four different NDDs (AD, PD, ALS, and HD) in this section. These four NDDs have remarkably different clinical features and pathogenic mechanisms, but vascular dysfunction is one of the common phenotypes for all of them. Leakage of the brain vessels can be observed in all these NDDs. Additionally, the pathological alterations in BBB cells are quite similar. TJ proteins reduction in ECs, pericyte loss, and displacement of astrocytes endfeet are reported in most NDDs. These results indicate that BBB disruptions are common features in NDDs and might participate in disease onset and progression. Thus, the BBB is considered one of the potential treatment targets for NDDs. From another point of view, treatments targeting the BBB may apply to all NDDs.

## 4. Human-Induced Pluripotent Stem Cells in Blood–Brain Barrier Modeling

iPSCs are generated from somatic cells. This breakthrough technology was published in 2006 by Yamanaka’s group and is now widely applied in biology research [183]. In brief, somatic cells (usually from a skin biopsy or a blood sample) from patients are reprogrammed by introducing a set of transcription factors: OCT4, SOX2, KLF4, and MYC, which are able to convert somatic cells back to stem cells. The transduction of transcription factors can be achieved by viral vectors, plasmid DNA, RNA, and recombinant protein delivery [183,184,185]. iPSCs can self-renew and differentiate into different cell lineages like other pluripotent stem cells. With appropriate stimulation and guidance factors, one can generate the desired cell type for the research. iPSC technology provides several advantages, including unlimited proliferative activity and the potential to form any human body cell. In addition, iPSCs are able to model human disease by reprogramming the somatic cells from patients harboring disease-related genes or introducing mutations via CRISPR gene editing [6,184,185,186].

Since 2007, the use of human iPSCs has increased tremendously, especially in the research of neurological diseases [187]. iPSCs have been successfully used to model several NDDs, such as AD, PD, ALS, and HD [188,189]. While most of these studies have focused on neuronal pathology, other brain cell types have gained interest, and publications in this area are expected to increase in the following years. iPSC-derived models have a great potential in drug screening applications and could potentially bridge the gap between preclinical and clinical studies in NDDs [190]. However, this field is still in the early days, and only a few studies have reported drug screening applications. In addition, most of these studies have only screened test or experimental drug candidates instead of large compound libraries.

In addition to the opportunities, iPSCs still have several limitations in NDD modeling and drug screening [189,190]. One of the problems is high variation, originating from different cell lines or clones, reprogramming, or differentiation protocols. Using isogenic controls or cells from cell banks could reduce the problem. In addition, more robust differentiation protocols are needed to reduce the heterogeneity in iPSC-derived cultures. Secondly, iPSC-derived cells have a juvenile phenotype, and age-related epigenetics are lost during the reprogramming. Some aging factors, such as progerin, have been applied to acquire more aged phenotypes in neurons. Finally, most of the studies have been done in 2D cultures with one cell type. These cultures lack cell–cell interactions and physical in vivo-like surroundings. Incorporating several cell types could help to reveal different disease-related pathways and recognize cell type-specific drug effects. Advances in forming brain organoids and microfluidic platforms could provide a solution to these limitations in the future.

Below, we summarize the progress and challenges of generating BBB-related cells from iPSCs and the different iPSC models that have been introduced into BBB research.

### 4.1. Differentiation of BBB-Related Cells

#### 4.1.1. Brain Endothelial Cells

The first protocol of iPSC-derived brain ECs was published in 2012 by Lippmann et al. [38]. This protocol relied on the co-differentiation of neuronal and brain ECs in unconditioned media for six days. Changing media to endothelial serum-free media supplemented with FGF and platelet-poor plasma-derived serum was used to expand the brain EC population. Finally, the brain ECs were purified by subculturing the brain ECs on collagen IV/fibronectin-coated plates. These brain ECs expressed TJs and nutrient transporters and had polarized efflux transporter activity. They also responded to astrocytic cues and had a high transendothelial electrical resistance (TEER) value (1450 ± 140 Ω × cm^2^). This protocol was modified in 2014 by adding retinoic acid to the original protocol to enhance the brain EC properties (expression of VE-cadherin and TJ proteins and increased TEER) [191]. Later, some additional modifications have been made, including the activation of WNT/beta-catenin signaling, resulting in higher TEER and lower batch-to-batch variation [192] and developing protocols with shorter time and defined conditions [193,194]. In addition to the protocols based on the original Lippmann publication, Praça et al. published a different protocol in 2019 [195]. The protocol relied on a two-step differentiation of brain capillary-like ECs from endothelial progenitors using VEGF, Wnt3, and retinoic acid induction. These cells expressed TJ proteins ZO-1 and claudin 5 but had a moderate expression of P-gp and TEER values (60 Ω cm^2^). Functionally, these cells responded to inflammatory stimuli by upregulating ICAM-1 expression after TNFα stimulation.

Since the publication of the original protocol by Lippmann and the subsequent modifications, these protocols have been widely adopted in the BBB modeling field. ECs generated with these protocols have shown several EC-like properties such as high TEER, tube formation, low-density lipoprotein uptake, and efflux transporter activities. The cells also express selective brain EC markers such as CD31, VE-cadherin, and claudin 5. Although the cells express several brain EC properties and markers, the characterization has been mainly focused on the in vitro barrier properties. Recently, some questions have been raised against the cell identity of the brain ECs derived through these protocols (discussed in more detail in [196]). First, brain ECs have a specific inflammatory phenotype. They restrict immune cell infiltration and express low levels of adhesion molecules in normal conditions [197]. In inflammatory conditions, brain ECs become activated, secrete pro-inflammatory cytokines and upregulate the expression of cell adhesion molecules. A study by Nishihara et al. showed that brain ECs differentiated through two established protocols (Lippmann et al. 2014 and Qian et al. 2017) did not express the full array of adhesion molecules [198]. Therefore, they concluded that these cells are not suitable to study immune cell interactions. Secondly, transcriptional analysis indicates a more epithelial than EC phenotype of these cells. Delsing et al. compared ECs derived through the neuro-endothelial protocol to vascular iPSC-derived ECs [199]. The transcriptomic analysis showed a decreased expression of EC-related genes in neuro-endothelial derived ECs. In contrast, several genes related to epithelial cell fate were expressed, including *CLDN1*, *CLDN4*, and *CLDN7*. The group concluded that these cells have a mixed phenotype. A more recent and detailed study by Lu et al. compared RNA expression of iPSC-derived brain ECs from previously published protocols to primary EC controls and iPSC-derived vascular ECs [200]. The data showed that iPSC brain ECs lacked the expression profile of ECs, and on the other hand, they expressed several epithelial genes (*EPCAM*, *KRT8*, *KRT19*, *SPP1*, and *FREM2*). The results were confirmed with single-cell RNA sequencing. The group concluded that even though the cells have tight barrier function, the cellular identity of ECs is missing, making them unsuitable for BBB in vitro modeling.

#### 4.1.2. Astrocytes

The differentiation of astrocytes is typically done through neuronal progenitor cells (NPCs). In 2011, Krencik and Zhang published a protocol where iPSCs are first differentiated to neuroepithelial cells and then collected and cultured as spheres in the presence of epidermal and fibroblast growth factors to produce astro-glial progenitors [201]. Finally, after a long-term expansion (90–180 days), the progenitors are maturated by dissociating the spheres and plating them down in the presence of ciliary neurotrophic factor. Additionally, the protocol allowed to generate different subtypes of astrocytes by adding specific morphogens in the neural epithelial phase. After that, additional protocols have been published with a shorter differentiation time [202,203,204,205]. As a downside, some of these faster protocols rely on the use of serum during the differentiation and culture. Serum might affect the reproducibility of differentiation and change the phenotype of astrocytes. This was emphasized in a protocol published in 2018 [206]. In this protocol, glial progenitors were first differentiated from neural stem cells, followed by astrocyte differentiation and maturation within 10–11 weeks. The addition of serum to the culture induced transcriptomic changes in the metabolism and astrocytes acquired a more reactive phenotype.

#### 4.1.3. Pericytes

Unlike neurons and astrocytes as specific cell types in the CNS, pericytes cover capillaries and venules throughout the body. Hence, the developmental origin of the pericyte is heterogeneous [207]; pericytes in different tissues might arise from other development layers. According to studies, CNS pericytes originate from two sources: mesoderm and neural crest [40,207,208].

To differentiate pericytes from iPSCs, most of the published protocols refer to the origin of pericytes and induce iPSCs to first differentiate toward mesoderm cells or neural crest cells [107,209,210,211,212]. The neural crest is induced through inhibition of the glycogen synthase kinase 3 (GSK3) and bone morphogenetic protein (BMP)/TGF-β signaling pathway, which is usually achieved by CHIR99021 (GSK-3 inhibitor) and SB431542 (BMP/TGF-β pathway inhibitor) treatment [209,210,211]. In contrast, the BMP-4 activating WNT/β-catenin pathway combined with GSK3 inhibition differentiates iPSCs toward mesoderm specification [107,209,212]. Alternatively, Kelleher et al. described a protocol to generate neural progenitors by BMP-4 and SB431542 rather than neural crest or mesoderm for further pericyte differentiation [213]. To ensure cells differentiate toward the right direction, some protocols positively select p75 neurotrophin receptor (p75NTR) expressing cells to isolate the neural crest cells, and some check by the immunostaining [210,211]. After generating the mesoderm, neural crest cells, or neuronal progenitors, these cells are treated with platelet-derived growth factor two B subunits (PDGF-BB) and TGF-β signaling for pericyte induction [107,211,212,213,214]. However, Kumar et al. claim that cells differentiated with PDGF-BB are more pericyte-like cells, while those induced with TGF-β 3 are smooth muscle-like cells, as evidenced by different marker expressions [212]. After pericyte induction, cells keep proliferating and are available to use for several passages. However, some protocols suggest using them only with a few passages, as the long-term culturing of pericyte-like cells may change their properties [215,216]. Kumar et al. described these proliferating cells as “immature” pericytes. They continue to mature cells with the PDGF-BB and BMP/TGF-β pathway inhibitor and with or without epidermal growth factor (EGF), giving them two different populations of cells. These two populations can be distinguished by the ligand expression level of CD274 (PD-L1) and DLK1, where the authors hypothesize that CD247 positive cells act as capillary pericytes while DLK1 positive cells act as arteriolar pericytes [212].

To characterize the generated pericytes, most publications check for classic pericyte markers such as PDGFRβ, NG2, CD13, CD146, and α-SMA through immunostaining or FACS [209,214,215,217] Some studies also examine EC marker expression levels such as CD34 and VE-cadherin to ensure that cells are not differentiating into other lineages [209,215]. However, these markers are not specific for pericytes only, and some might also be expressed in astrocytes and ECs [208,217]. As such, Faal et al. also investigated the expression level of FOXF2, VTN, and FOXC1, which are required for pericyte differentiation in humans [209]. Comparisons of the RNA sequencing results between iPSC-derived pericytes and pericytes from the human brain have been performed in a couple of studies [210,212]. These results showed that iPSC-derived pericytes are similar to the pericytes from brain tissues and function normally to support endothelial function. This makes iPSC-derived pericytes an excellent tool for investigating pericytes’ function in healthy and diseased brain. Another important way to characterize the generated pericytes is to check if they function properly. Mouse pericytes co-cultured with human ECs improve the integrity of the ECs [42], and this phenomenon is also observed in ECs cultured with iPSC-derived pericytes. The TEER value is increased, and endothelial elongation while co-culturing with iPSC-derived pericytes indicates that pericytes improve and regulate their function [209,210]. In addition, these iPSC-derived pericytes can arrange in a tube formation, indicating angiogenesis ability, or they self-assemble a vessel-like structure in 3D culture with ECs [209,210,212].

Although iPSC-derived pericytes have many benefits, such as the unlimited number of cells and the ability to create humanized models, they still have some remaining problems. While studies have tried to identify pericytes by combining many genes at the expression level, it is still difficult to distinguish pericytes from smooth muscle cells [208,217]. In addition, comparison with pericytes from tissues may not be that reliable because the isolated cells could be a mixed population of mural cells or other perivascular cell types. Hence, transcriptomic or proteomic studies should be performed to distinguish pericytes from smooth muscle cells before further in vitro studies take place.

### 4.2. Blood–Brain Barrier In Vitro Models

Since ECs, astrocytes, and pericytes are required for establishing a fully functional BBB, co-cultured models are developed to mimic the BBB formation. Additionally, to better understand the cell interactions between BBB cell types, more complicated 3D models are invented. Here, we reviewed several BBB models that apply iPSC-derived ECs, astrocytes, and pericytes into the co-culture system. Each model has its advantages and limitations. The researcher can decide which model to use according to the research objectives.

#### 4.2.1. Transwell

Transwell is a common model for co-culture experiments. Briefly, insert membranes with tiny pores are placed on the top of the usual culture plates. ECs can be plated on the apical side of inserts and other cell types such as astrocytes and pericytes can be plated in lower compartments (culture plates) to achieve non-contact co-culture. Contact co-culture can be done by seeding cells on both sides of inserts, and cells may have physical contact through the pores on insert membranes [218]. As a simple and straightforward way to establish the BBB model, the transwell has many advantages and is available for iPSC-derived cells. First of all, it is fast and cost-effective. Unlike 3D co-culture or organoids that may need to grow cells for several weeks to months, cells re-plated on the transwell could be harvested for functional testing within a few days [219]. These properties also make the transwell model more suitable for genotype or drug screening. Second, it is applicable for multicellular co-culture and can be used to observe the difference between contact or non-contact culture to investigate if the phenotype is coming from a physical contact [218]. The permeability assay can be conducted on the transwell model to determine the endothelial integrity, which is otherwise not easy to observe in 3D culture or organoids [218,220]. Lastly, the transwell model is able to test the activity of transporters on the ECs. Studies have already shown that expression levels of transporters such as P-gp 1 are downregulated in AD patients. The transwell can provide evidence of the functional degradation of transporters, other than by measuring gene expression levels [220,221]. The transwell model has some limitations as well. First, although different cell types may have physical contact with each other in the transwell model, it is still a 2D culture model that lacks 3D organization of the vessels’ structure in the human body. This makes the transwell model unable to mimic direct cell–cell interactions in the BBB, making cells lose some of their properties, such as ligand–receptor mediating response [219]. Second, blood flow in the vessels causes mechanical forces on the ECs termed shear stress. Studies have revealed that iPSC-derived ECs manifest a unique phenotype when responding to shear stress, such as proliferation rate and cytoskeleton re-organization. That is to say, without the shear stress to the ECs, ECs also lose some of their properties [222,223]. Last, it is challenging to image cells from both sides of the transwell, so it may not fit those experiments that need to track cell morphology changes.

Despite the disadvantages mentioned above, studies that establish a BBB on the transwell model have brought some important results (Table 1). In the first place, iPSC-ECs were co-cultured with human or murine primary astrocytes and pericytes. Increased TEER values can be found in both co-culture (ECs with astrocytes or pericytes) and complete BBB culture (ECs with astrocytes and pericytes) compared to ECs monoculture. The TJ proteins expression level is also elevated in BBB culture in contrast with ECs monoculture [38,191,192,193]. These results indicate the importance of pericytes and astrocytes in improving the barrier integrity and regulating ECs function. Later on, with the development of iPSC-derived astrocytes and pericytes, the transwell model was used to verify iPSC astrocytes and pericytes from new protocols. TEER was the most common tool to decide whether iPSC astrocytes and pericytes have the ability to enhance barrier function similar to primary astrocytes and pericytes [194,199,209,210]. Some publications also show the increased TEER value in iPSC-ECs co-cultured with neurons, suggesting that neurons may be able to regulate BBB functions [191,210,224]. Moreover, both contact and non-contact co-culture of ECs, astrocytes, and pericytes show improvement of ECs integrity, which implies the secretion of molecules to communicate between cell types [107,215]. The transwell model has also been applied to study the permeability of different sizes of molecules, which can help predict the permeability of drugs to the brain [225]. Although drug permeability can be quickly screened by ECs monoculture, a complete BBB model provides more accurate results and saves effort before commencing an in vivo study.

#### 4.2.2. Spheroid

Spheroid is a 3D culture model where cells are cultured in low-adherence conditions [226]. Multiple cell types can be cultured together, allowing interactions between the cell types and self-assembling of the cells into a tissue-like structure [227]. Unlike in many other models, all essential cell types of the tissue can be cultured in the same spheroid to make the most accurate tissue model possible [228]. Spheroids can be used to study organogenesis [229], the permeability and function of different drugs, neurotoxicity, and disease modeling [230,231]. The spheroids can be cultured for several weeks, so they can also be used to study the long-term effects of drugs.

Assays that can be done for spheroids are, for example, permeability assays, immunofluorescence staining, transporter activity assay, and flow cytometry [227,228,230,232]; however, TEER cannot be measured. In addition, permeability tests can be made for spheroids only once instead of transwells and organ-on-chips that can be made many times to follow the barrier integrity. Despite their possibilities, the spheroids with iPSC-derived cells have not been used for modeling BBB. As a lack of publications of spheroid BBB iPSCs models, we do not focus on this model as much as on other models.

#### 4.2.3. Organ-on-Chip

Organ-on-chip is one of the newest in vitro models. It enables more realistic modeling of tissues and organs by mimicking the microenvironment in vivo. In organ-on-chips for BBB modeling, the 3D structure resembles the structure of blood vessels, and flow can be generated through the channel to simulate blood flow and to subject the ECs layer to shear stress [233,234,235]. The basic structure of the organ-on-chip includes at least two cell culture chambers. The first chamber is a channel with open ends, and the second cell culture chamber can be a channel or an open-top chamber, separated from the first chamber by a porous membrane. ECs are plated in the first cell culture channel and the other cell type(s) to the second channel or open-top chamber. The porous membrane between the cell culture chamber enables the transfer of substances and interactions between ECs and other cell types, similarly to transwells [236,237,238,239,240]. However, the chip structure can have a lot of variation due to different requirements of the users; for example, a membrane is not always used in chips, and there may be more than two cell culture chambers and additional medium channels. Similar to transwells, organ-on-chip is a fast model to make and use for analysis. ECs form the barrier in a few days after re-plating.

Organ-on-chips may cause problems for some analysis methods compared to transwells. For example, qPCR is more difficult to perform due to the smaller cell amount, and the measuring of TEER is possible only with integrated sensors. Instead, for example, live-cell imaging is possible for the chip, unlike for the transwells. The most used analysis methods for organ-on-chips include live and dead cell imaging, different permeability assays, fixation, and further analysis such as immunofluorescence staining and TEER measurement. In addition to the measuring of TEER, the integrated electrodes can be used for measuring shear stress in the channels.

Organ-on-chips have become an attractive choice for BBB modeling because they can stimulate the flow and shear stress and better mimic the BBB microenvironment than other models [234]. However, organ-on-chips are still in the early stage of development, and several challenges need to be addressed before they can be widely used in the BBB field. Usually, organ-on chips are made of polydimethylsiloxane (PDMS), which is bio-compatible, optically transparent, flexible, non-toxic, and gas-permeable material. It is also easy to mold and cost-effective. However, PDMS can absorb hydrophobic molecules and thus may cause biases to the results [241]. Additionally, the fabrication and handling of the chips still require technical skills end special equipment which is not necessarily available for all the labs [234]. Several organ-on-chips also have a porous membrane to separate the different cells [242]. These membranes are often thicker compared to the basement membrane in ECs and prevent direct cell–cell contact. Despite the challenges, the use of organ-on-chips is expected to increase, and new developments could resolve some of the issues.

Several different BBB models with iPSC-derived cells have been established in various studies. However, there are many differences—for example, in used organ-on-chip platforms, cells, culture conditions, membranes, and methods for generating the flow through it. Some of the studies are made only with iPSC-derived brain ECs [236], while others have used iPSC-derived astrocytes [239], neural cells [237,240], or primary astrocytes and pericytes [237,238,240]. more specific information on these studies and their differences has been collected (Table 2).

#### 4.2.4. Hydrogel Models

Hydrogels are one type of 3D cell culture model to enable more physiologically relevant modeling of tissues. In hydrogel models, several cell types can be cultured in close proximity to each other, and the cells can self-assemble to form 3D structures that resemble those in a live organism. As in spheroids, there is no physical limitation in physical interactions between cell types [247]. Different imaging techniques can be used to monitor the changes in the culture. In addition, permeability and transporter activity assays can be performed. Hydrogels can also be used as part of the other models [239].

Hydrogels can be made of synthetic and/or natural polymers to resemble extracellular matrices. The properties of the synthetic hydrogels, such as polyethylene glycol and polyacrylamide, can be tuned by changing molecular weights or crosslinking degrees, and the composition of batches has very little variation. However, they lack bioactive components such as cell adhesion motifs that might affect the behavior and function of the cells. Instead, hydrogels made of purely natural polymers can have these bioactive ECM components. Still, their mechanical properties are not as good as synthetic polymers, and there is variation between batches. Composite hydrogels with several components, synthetic and natural, have been developed to solve these problems [247].

BBB models on hydrogels using iPSC-derived cells are limited. These models and their details are shown in Table 2. Faley et al. (2019) produced a hydrogel BBB model with iPSC-derived ECs [244]. The model they used was similar to organ-on-chip but with only one channel and made of a hydrogel consisting of gelatin and transglutaminase. A pump was used to generate the flow through some of the models, while others were maintained in static conditions for comparison.

The hydrogel was also used in a study by Campisi et al. (2018) [243]. The study utilized iPSC-derived ECs, primary astrocytes and pericytes, plated into three channels. The astrocytes and pericytes were plated into the middle channel in fibrin hydrogel, allowing cells to self-assemble. The other two channels, located on both sides of the middle channel, were plated with iPSC-derived ECs. However, no continuous flow through the channels was generated.

#### 4.2.5. Vascularized Brain Organoids

Since the original Lancaster protocol, the use of brain organoids has advanced rapidly. Importantly, organoids have been adapted to disease modeling in several NDDs. While the organoids have several advantages compared to other models—expected cell–cell interaction, several cell types present, and spatial organization—they still have many shortcomings [248]. Mainly, not all cell types are present in organoids, and the lack of vascularization causes a necrotic core to form. In addition, high batch-to-batch variation and technical challenges make organoids unsuitable for high-throughput screening purposes. To address the lack of vascularization, studies have now developed organoids with vascular-like systems. Pham et al. generated vascularized brain organoids by adding iPSC-derived ECs to the organoids on day 34 [245]. Vascularization of the organoids was seen after 3–5 weeks. In another study, Cakir et al. used human embryonic stem cells expressing human ETS variant 2 (hETV2) to create a vascular system in cortical organoids [246]. The expression of ETV2 resulted in a vascular-like network in the organoids and enhanced the functional maturation of neurons. Moreover, the BBB characteristics were detected in these organoids, including an increased expression of TJ proteins, nutrient transporters, and TEER (Table 2). The results demonstrate the possibility of incorporating a vascular network to brain organoids, and these types of models could potentially be valuable sources for disease modeling in the future.

iPSC-derived BBB models have several advantages compared to other in vitro systems. As described previously, iPSC has unlimited sources of cells and can turn into any cells of our body with appropriate guidance factors [186]. The primary brain cells from human tissues are hard to obtain and have a limited lifespan. In contrast, primary animal cells are easier to get but may not accurately mimic human cell response. The immortalized cell lines also have issues in performing as ‘in vivo’ cells [249]. Due to these reasons, iPSC-derived cells have become the most potent tool in this field. From another aspect, the iPSC model is more suitable for disease modeling. Unlike cell line or primary animal cells only harboring one or two mutation genes, iPSC cells are acquired from patients who may carry several genetic risks [250,251]. Many studies have claimed that AD, PD, and ALS might be polygenic [252,253,254], and thus, iPSC-derived models from these patients may closely mimic the actual pathologic phenotype of these diseases. In addition, iPSCs can be used to build more complex models, such as organoids. Organoids are similar to a tiny brain on the dish where iPSCs grow and differentiate toward several brain cell types simultaneously [245,246]. Although the vascularized organoids are still in the developing stage, it looks promising to build a model containing vasculature and neurons. With the organoids, we can observe the interaction of BBB cells and other cell types and link, for example, the evidence between vascular dysfunction and neuroinflammation by this model.

## 5. iPSC–BBB Models of Neurodegenerative Diseases

### 5.1. Alzheimer’s Disease

**Figure 1:**
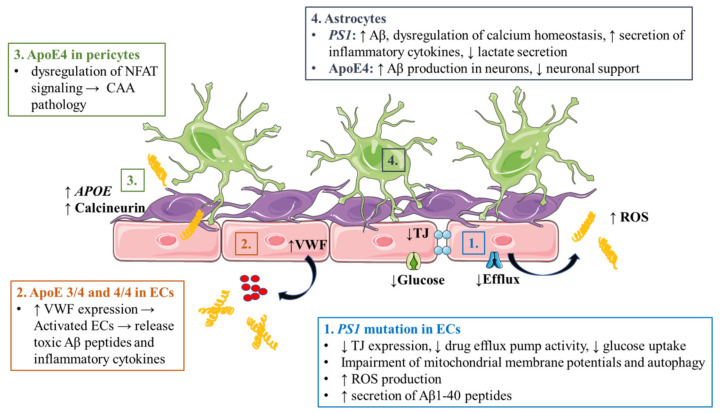
Schematic illustration of BBB dysfunction in AD based on iPSC-BBB models.

Since neuron loss is one of the major characteristics of NDDs, most iPSC studies focus on neuronal cell pathology. However, with more and more evidence indicating the contribution of cerebrovascular dysfunction in AD progression, the investigation of cell types forming brain vasculature is becoming more critical. Several studies have described the defects in iPSC-derived ECs with the PS1 mutation, a genetic risk factor for familial AD. Impaired barrier function resulting from downregulated TJ protein expression, reduced drug efflux pump activity, and glucose uptake was reported in these studies [221,255,256,257]. In addition, impairment of the mitochondrial membrane potentials and autophagy were also discovered in PS1 mutation ECs [257]. One study also detected increased ROS production and elevated secretion of Aβ1-40 peptides from ECs harboring the PS1 mutation [255]. Defects have also been observed in ECs carrying ApoE4 alleles, the major risk for sporadic AD. Those ECs with the ApoE 3/4 or 4/4 genotype are found to overexpress platelet-binding protein VWF, which relates to EC activation. Activated ECs turn into a pro-inflammatory state and release more toxic Aβ peptides as well as cytokines [258] (Figure 1). These results suggest that minor defects accumulated in ECs eventually lead to BBB dysfunction. ECs harboring mutations associated with AD are sufficient to independently cause these defects.

Some studies, including ours, have generated iPSC-derived astrocytes with AD-associated mutations to reveal the importance of astrocyte pathology in AD. In our research, PS1 mutant astrocytes show increased Aβ production, dysregulation in calcium homeostasis, and increased secretion of inflammatory cytokines. Additionally, lactate secretion is reduced, influencing the crosstalk between astrocytes and neurons [89]. Culturing neurons with conditioned medium from iPSC-derived astrocytes carrying ApoE4 alleles increased the Aβ production in neurons [259]. Directly co-culturing neurons with astrocytes show that ApoE4 astrocytes are less effective in promoting neuron survival and synaptogenesis than those of ApoE3 astrocytes [260]. From these studies, we could assume that astrocytes harboring AD-related mutations may also contribute to BBB dysfunction by increasing Aβ production or releasing inflammatory factors (Figure 1). However, more studies still need to identify the role of astrocytes in the BBB and how AD astrocytes contribute to cerebrovascular dysfunction.

Several studies have tried to combine all cell types of the BBB in transwell, organ-on-chip, or 3D co-culture models. However, most publications were optimizing the methodologies or differentiating protocols. Therefore, there is still a lack of studies on a complete iPSC-derived BBB model in AD. Recently, researchers cultured iPSC-derived ECs, astrocytes, and pericytes in Matrigel to form a 3D assembly BBB model (in the following text called iBBB). In this study, they observed more amyloid accumulation in ApoE4 iBBB than ApoE3. By combining ApoE3 or ApoE4 ECs, pericytes, and astrocytes, they found that ApoE4 pericytes are required for this phenotype. Their experiments reveal that dysregulation of the calcineurin-nuclear factor of activated T cells (NFAT) signaling in ApoE4 pericytes induced the CAA pathology in the iBBB model [107].

### 5.2. Parkinson’s Disease

**Figure 2:**
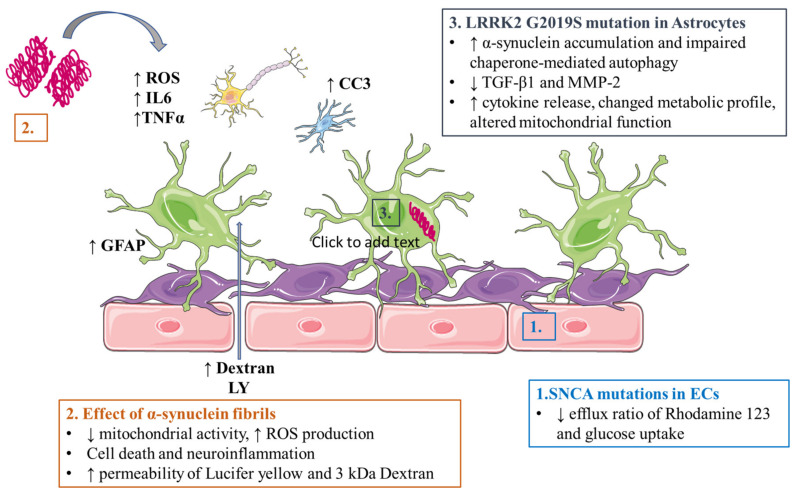
Schematic illustration of BBB dysfunction in PD based on iPSC-BBB models.

In PD, the research focus has been mainly on DA neurons. However, interest in other brain cells has increased recently. So far, only one study has been published describing the role of PD-related mutations in iPSC-derived ECs. In this study, the effect of two SNCA mutations (A53T and triplication) on barrier properties was examined [256]. Both mutations reduced the efflux ratio of Rhodamine 123, suggesting reduced functionality or an incorrect polarization of efflux transporters. Additionally, SNCA triplication ECs showed decreased glucose uptake (Figure 2). While this study suggested that PD-related mutations can disrupt the barrier function, the study included only one line per mutation. It is clear that more studies are needed to assess the role of different PD-related mutations in ECs.

A few studies have now investigated the role of PD-related mutations in iPSC-derived astrocytes. The first study showed increased α-synuclein accumulation and impaired chaperone-mediated autophagy in LRRK2 G2019S astrocytes [261]. Additionally, LRRK2 mutated astrocytes induced morphological changes to healthy neurons. In another study, LRRK2 G2019S astrocytes downregulated TGF-β and MMP-2, suggesting reduced neuroprotective capacity [262]. In our study, iPSC-derived astrocytes obtained from LRRK2 G2019S mutant patients with one patient also carrying the GBA N370S mutation showed several PD-related features, such as increased α-synuclein levels, more reactive phenotype after inflammatory stimuli, changed metabolic profile, and altered mitochondrial function [263] (Figure 2). Although it is clear that astrocytes have an important role in PD, how PD astrocytes affect the BBB function has still not been studied with iPSC-derived cells.

Recently, a new study assessed α-synuclein pathology using a human substantia nigra brain chip [240]. The chip was constructed with two channels separated by a porous membrane. Human iPSC-derived dopaminergic neurons with primary astrocytes, pericytes, and microglia were cultured on one side, and iPSC-derived ECs were cultured on the other side of the membrane. After the characterization of the model, the brain side of the chip was exposed to α-synuclein fibrils. This led to reduced mitochondrial activity and increased ROS production. Additionally, α-synuclein fibrils induced cell death and neuroinflammation. The effect of α-synuclein on the BBB was also studied. Fibrils caused increased permeability of Lucifer yellow and 3 kDa Dextran on day 8 of the culture, indicating disruption of the BBB. RNA sequencing revealed several altered pathways in the chip exposed to α-synuclein fibrils (Figure 2). These included upregulation of inflammation, autophagy, mitochondrial oxidation, vesicular trafficking, endothelial efflux, and lipoprotein receptors. Downregulated pathways included solute carrier-mediated transports, TJs, and gap junctions.

### 5.3. Amyotrophic Lateral Sclerosis

**Figure 3:**
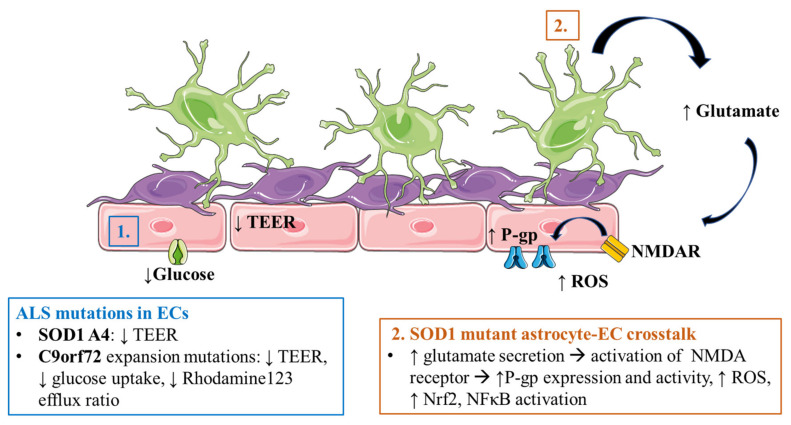
Schematic illustration of BBB dysfunction in ALS based on iPSC-BBB models.

For ALS, the effect of SOD1 A4 V and C9orf72 expansion mutations on barrier properties in iPSC-derived ECs was investigated in one study [256]. Both mutations significantly reduced TEER values in ECs and the C9orf72 mutated cell line had decreased glucose uptake and Rhodamine 123 efflux ratio (Figure 3). These results suggest that ALS-related mutations might affect the EC function, but more studies are needed to assess the role of different mutations.

The role of astrocytes in ALS has been widely recognized. Several studies have used iPSC-derived astrocytes to demonstrate the ALS-related phenotype and their ability to cause non-cell-autonomous pathophysiological changes in motor neurons [202,264,265,266,267,268,269]. In addition, a few studies have explored how ALS mutant astrocytes affect the EC properties. The first study showed that SOD1 mutant astrocytes upregulated P-gp expression in ECs, which was associated with increased ROS production, Nrf2, and NFκB activation [270] (Figure 3). A follow-up study examined the mechanisms in more detail and found that excess glutamate secreted from SOD1 and sporadic ALS astrocytes drove the upregulation and activity of P-gp in ECs via the activation of N-methyl-D-aspartic acid receptor [271]. Interestingly, the mechanism was not shared with all forms of ALS. C9orf72-ALS astrocytes did not secrete higher levels of glutamate and did not affect the P-gp expression in ECs. These studies highlight the complex interplay between different ALS astrocytes and ECs, which could impact disease prognosis and the efficacy of pharmacotherapies.

### 5.4. Huntington’s Disease

**Figure 4:**
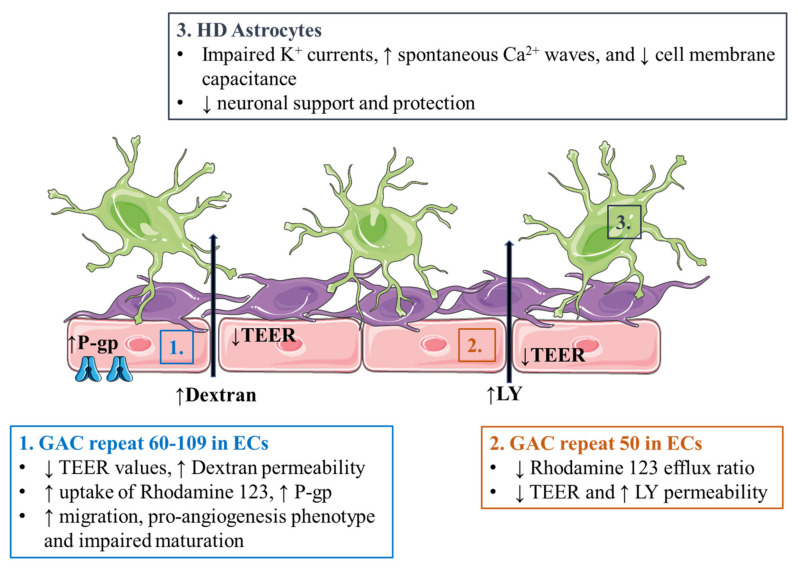
Schematic illustration of BBB dysfunction in HD based on iPSC-BBB models.

Three studies have now described the effect of different lengths of CAG repeats on iPSC-EC function. The first, and more extensive study, described several changes in HD iPSC-ECs [272]. The study included HD lines with expanded repeats of 60Q, 66Q, 71Q, and 109Q. The changes included functional deficits in angiogenesis and barrier properties. The HD ECs had increased migration and decreased TEER values. Additionally, the HD ECs had increased uptake of Rhodamine 123 and increased MDR1 levels, suggesting altered MDR1 function in HD ECs (Figure 4). RNA sequencing found altered pathways in HD ECs, including WNT/β-catenin, pro-angiogenic, and leukocyte extravasation signaling pathways. In addition, genes promoting vascular sprouting and remodeling were altered in HD ECs. The data suggest a pro-angiogenesis phenotype of HD ECs together with impaired maturation of the cells. The effect of HD mutations in iPSC-ECs was also examined in another study [256]. The study included lines with CAG 50 and 70 repeats. Both mutations decreased the Rhodamine 123 efflux ratio, suggesting altered efflux transporters. The HD50 line also had decreased TEER and increased permeability of Lucifer yellow (Figure 4). Interestingly, in the previous study, the decrease of TEER was more profound in the lines with longer repeats. Vatine et al. demonstrated that BBB organ-on-chip could be a relevant system for disease modeling [237]. The permeability of different size dextrans was examined in the BBB chip derived from an HD patient with 71 CAG repeats and compared to healthy ones. The permeability was significantly increased in the HD BBB chip. It is good to bear in mind that all the studies only used one line per mutation, and more studies are needed to assess the effect of different lengths of CAG repeats on EC function.

Although no studies examine how HD astrocytes affect the EC or BBB function, HD iPSC astrocytes have been shown to recapitulate an HD-related phenotype. Astrocytes from individuals carrying the 50 or 109 CAG repeats had cytoplasmic, electron clear vacuoles, a phenotype observed in peripheral blood lymphocytes in HD [273]. Another study with HD astrocytes (one line with 109 and 119 CAG repeats, one line with 77 CAG repeats) displayed several hallmarks of HD, including impaired inward rectifying K^+^ currents, lengthened spontaneous Ca^2+^ waves, and reduced cell membrane capacitance [274]. Additionally, HD astrocytes failed to support the maturation of iPSC-derived neurons and did not protect the neurons against exposure to chronic glutamate stimulation (Figure 4).

## 6. Discussion and Conclusions

Although there is already plenty of evidence linking neurovascular dysfunction to NDD progression, the underlying mechanism is not fully understood. For example, TJ proteins reduction and transporter activity downregulation are found in NDD brain ECs. Studies propose MMP-9 accumulation as a potential pathology [73,74], but how MMP-9 influences these proteins remains unclear. TJ proteins and ECM-related gene expression levels are also altered in ECs while co-culturing with pericytes [42,105], but the mechanisms are still not well understood. To solve these questions, transcriptomic and proteomic analysis tools need to be applied in this field. By comparing the cell profile between disease and control groups, we may know better which pathway is altered in disease conditions and contributes to vascular dysfunction. RNA sequencing analysis has been applied to neuronal cells and glial cells in many studies and provides some new perspectives in research [275]. Nevertheless, many studies do not focus on the vasculature cells and their changes in disease models so far.

Since neurovascular dysfunction is related to NDD progression and could be a potential therapeutic target, it is important to establish a stable model for drug development. The mouse model is often used for observing the phenotype and investigating the mechanism, but it is inadequate for drug screening. There are likely differences between the human and mouse BBB, such as the permeability to molecules and the response to drug treatment. Since drug screening requires a simple and fast readout to screen many compounds, the human iPSC models are necessary and can provide clear advantages in BBB studies and drug development.

In this review, we go through the protocols generating BBB-related cells from iPSCs and different models. Evidence supports that astrocytes and pericytes are crucial for EC function, including maintaining BBB integrity and regulating some gene expression levels, which is consistent with animal studies. In the disease model, the defects can appear independently in ECs, astrocytes, and pericytes carrying disease-related gene mutations but also affect other cell types when co-cultured with each other. Minor changes accumulate in all cell types and may eventually lead to the BBB breakdown. Despite successfully building the BBB model with iPSCs, some problems still need to be solved: first of all, the purity and accurate generation of cell populations. In fact, these problems might exist in most studies using iPSCs as a model. Although studies have tried to optimize the protocol to make it close to the actual situation in vivo, it is hard to know the precise induction pathway in the human developmental stage. Therefore, generated cells might have some properties of the desired cell types but are not fully functional due to the lack of stimulations during differentiation.

As discussed, iPSC-derived brain ECs are a prime example; almost all protocols can produce ECs expressing TJ proteins and perform the barrier function. However, some of the generated ECs are found to lack EC-related gene expression, such as CD31. In addition, the transcription profile analysis reveals that these ECs lack some EC properties, so it may not be suitable for studying the cell interaction with pericytes and astrocytes [196,200]. Nishihara et al. published a modified protocol claiming that their ECs can be used to investigate the interaction of the immune cells with ECs due to the constitutive cell surface expression of ICAM-1 and E-selectin, which are not found in ECs generated from previous protocols [198]. These results indicate that insufficient understanding of human brain EC development results in not generating more in vivo-like cells for further studies. Similar issues also happen in iPSC-derived pericytes. We used the term ‘pericytes’ in all our texts to avoid confusion. Still, in some publications, they were described as mural cells or pericyte-like cells because authors cannot distinguish smooth muscle cells from pericytes in their protocols [107,210,214]. Although some publications examine pericytes by marker expression, those markers are positive in both smooth muscle cells and pericytes, so we cannot exclude that there might be a mixed population of cells in the culture. As mentioned above, transcriptome and proteomics studies need to be introduced in this field to help clarify the difference between these cells. The more we understand these cells, the higher chance we could create a more accurate model for future studies.

The second problem is the complexity of the BBB. The BBB is not just composed of multiple cells but also subjected to shear stress due to the blood flow. Studies show that shear stress can mechanically stimulate ECs and influence cellular functions, including vascular remodeling and permeability, which means that ECs may act differently with and without shear stress [222,223]. Some organ-on-chips are able to mimic blood flow and have shear stress on ECs but may not be suitable to study the cell interaction and gene expression levels in this model. Spheroids and self-assembly 3D models in hydrogels are excellent for observing the vasculature changes and cell interaction phenotype but are not allowed to detect the BBB integrity changes. Every model has its advantages and limitations, and we cannot gather all the information from one model. The model used in the research depends on the questions addressed. Hence, we still lack one perfect model that solves all these problems and can be applied in all circumstances. Another defect in the iPSC BBB model is that we cannot link vascular dysfunction to neurodegeneration directly. Despite the alterations that appear in iPSC BBB with NDDs-related mutations, neuronal cells or microglia cells still need to be introduced to the model to check whether changes in vasculature indeed lead to neuroinflammation and degeneration. It is difficult to include so many cell types in the co-culture system, and a better iPSC model needs to be introduced to answer this question. However, with this completed model, we could study how exactly vascular dysfunction affects neuronal cells and immune cells and how it leads to, for example, dementia and motor neuron defects.

Despite these challenges, the iPSC-derived BBB model is still necessary and important for future research. With the established model, we could ask: (i) Could the BBB be the therapeutic target for NDDs? (ii) Can drug treatment that improves BBB function or stabilize vasculature structure prevent disease development? Since most cases of NDDs are sporadic and may relate to aging, lifestyle factors, and the environment, these could be the direction for future studies, and the iPSC model can be applied to these studies as well. Naturally, all of these studies are performed hoping that we could find a drug that prevents or even reverses the neurodegenerative processes to cure NDDs.

## Figures and Tables

**Table 1:** Blood–brain barrier in vitro models (transwell).

Citation	Experiment Conditions *	Main Readouts **
Lippmann et al. 2012 [38]	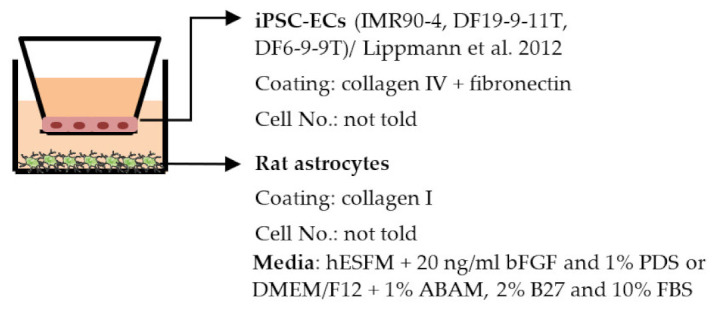	**TEER (Ω × cm^2^)** Monoculture: 222 Co-culture: 1450, (IMR90-4), 777 (DF19-9-11T)
Lippmann et al. 2014 [191]	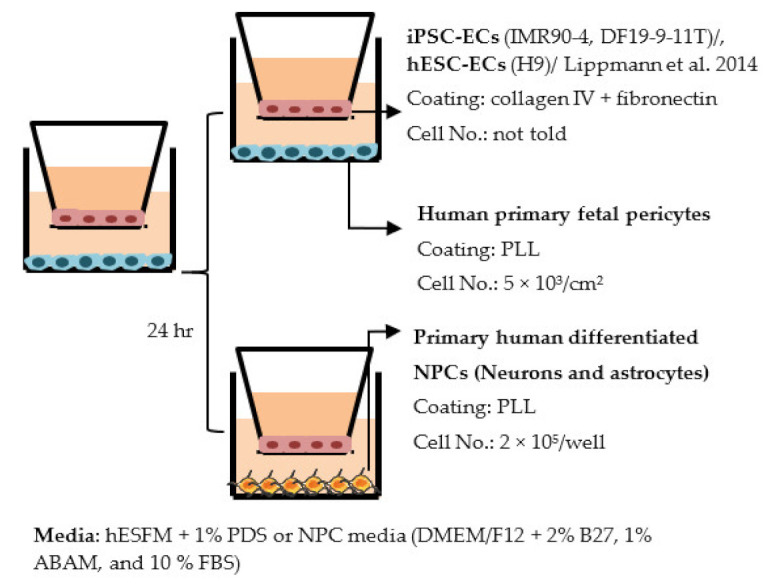	**TEER (Ω × cm^2^)** Monocultures: 3670 (IMR90-4), 1970 (DF19-9-11T), 1030 (hESC) Co-cultures: IMR90-4-ECs: 4450 (+pericytes), 5350 (+pericytes and NPCs) DF19-9-11T-ECs: 4740 (+pericytes and NPCs) hESC-ECs: 1680 (+pericytes and NPCs)
Qian et al. 2017 [192]	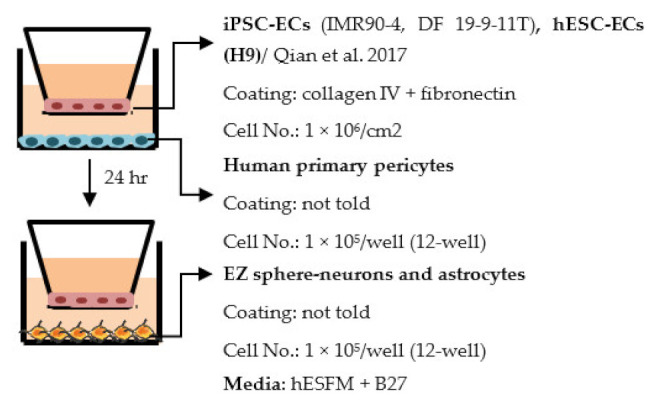	**TEER (Ω × cm^2^)** Monocultures: 3315 (IMR90-4), 1980 (H9), 3571 (DF19-9-11) Co-culture: ~30% increase compared to monoculture
Hollmann et al. 2017 [193]	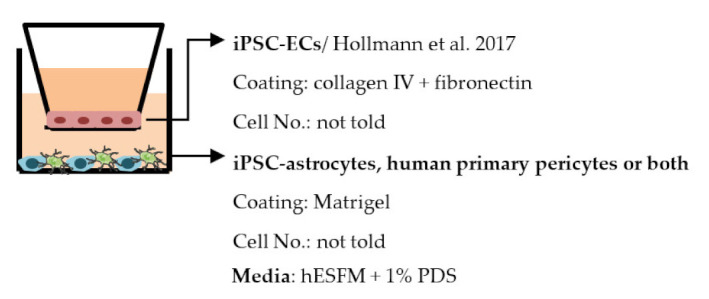	**TEER (Ω × cm^2^)** Monoculture: 4227 Co-cultures: 5378 (+astrocytes), 5937 (+pericytes), 6635 (+astocytes, pericytes) **Permeability, NaF (Pe × 10^−7^ cm/s)** Monoculture: less than 1.97 in all lines
Appelt-Menzel et al. 2017 [225]	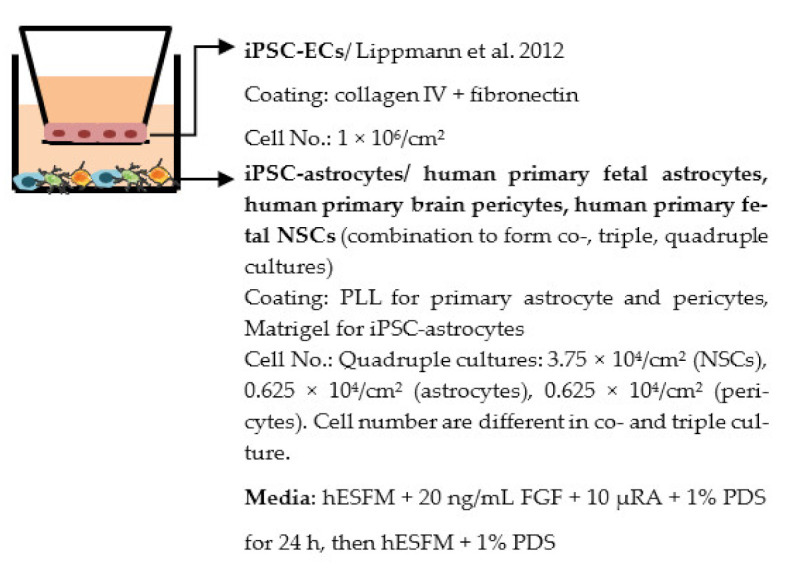	**Max TEER (Ω × cm^2^)** Monoculture: 2073 Co-cultures: 2024 (+primary astrocytes), 2485 (+iPSC-astrocytes), 2107 (+pericytes), 2171 (+hiPS-NSCs) Triple cultures: 2105 (+primary astrocytes, pericytes), 2293 (+primary astrocytes, hiPS-NSCs), 2556 (+pericytes, hiPS-NSCs) Quadruple cultures: 2489 (+primary astrocytes, pericytes and hiPS-NSCs) **Permeability coefficient (µm/min)** Monoculture: 1.52 (LY), 1.53 (fluorescein), 0.0166 (Dextran, 4 kDa), 0.0054 (Dextran, 40 kDa) Quadruple culture: 1.58 (LY), 1.33 (fluorescein), 0.0106 (Dextran, 4 kDa), 0.0030 (Dextran, 40 kDa) **Other** ↑ SLC2A1 in quadruple culture
Canfield et al. 2017 [224]	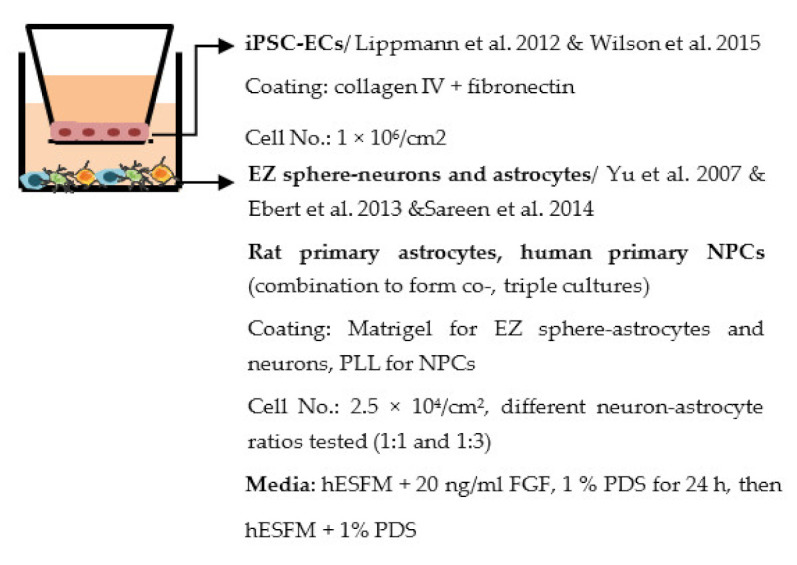	**TEER (Ω × cm^2^)** Monoculture: 153 Co-cultures: 491 (+neurons), 558 (+astrocytes), 611 (+NCPs), 784 (+primary astrocytes) Triple cultures: 661 (+neuron − astrocytes, 1:1), 886 (+neuron − astrocytes, 1:3) **Permeability, NaF (Pe × 10^−7^ cm/s)** Monoculture: 4.8 Co-cultures: 2.0 (+NCPs), 1.9 (+primary astrocytes) Triple cultures: 1.20 (+ neuron − astrocyte 1:3) **Other** Triple culture (neuron − astrocyte 1:3) increased TJ localization in iPSC-ECs, no change in transporter expression
Delsing et al. 2018 [199]	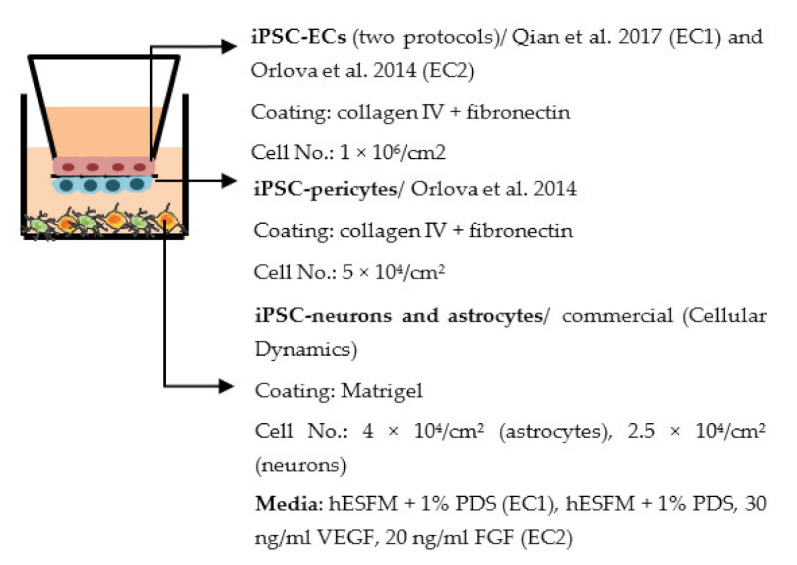	**TEER (Ω × cm^2^)** Monoculture: 773 (EC1), 52 (EC2) Co-culture: 1267 (EC1), 150 (EC2) **Permeability** Decreased NaF permeability in co-culture (EC1), exact values not told **Other** Changed gene expression in iPSC-ECs after co-culture: ↑ BCRP (EC1, EC2), ↑ P-gp (EC1), ↑ Glut1 (EC2), ↓ VE-cadherin (EC1), ↓ Caveolin 1 (EC1, EC2), ↑ occludin (EC2)
Neal et al. 2019 [194]	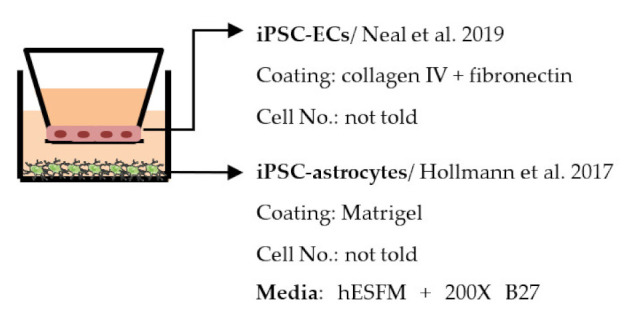	**Max TEER (Ω × cm^2^)** Monoculture: 8734 Co-culture: above 9000 **Permeability, NaF (Pe × 10^−7^ cm/s)** Monoculture: less than 2.5
Stebbins et al. 2019 [210]	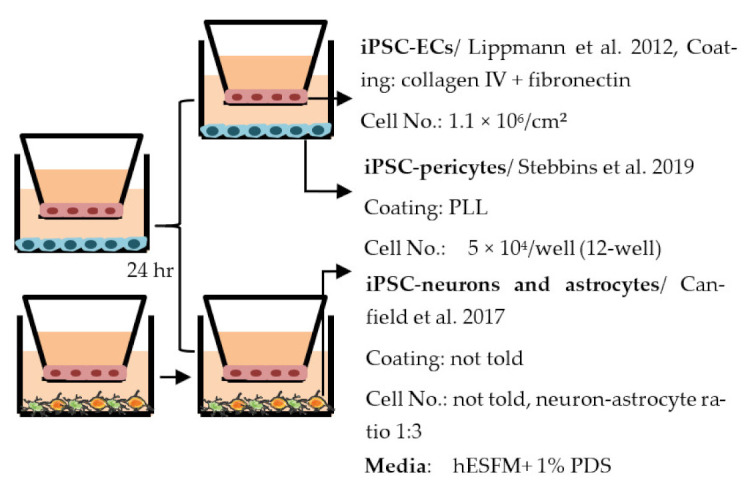	**TEER (Ω × cm^2^)** Monoculture: ≈300 (exact values not shown) Co-culture: 720 (+neurons and astrocytes), 503 (+pericyte), 1156 (+pericytes, astrocytes, and neurons) **Permeability (Pe × 10^−6^ cm/s), NaF** Monoculture: ≈6 Co-cultures: Five-fold decrease (exact values not shown)
Jamieson et al. 2019 [215]	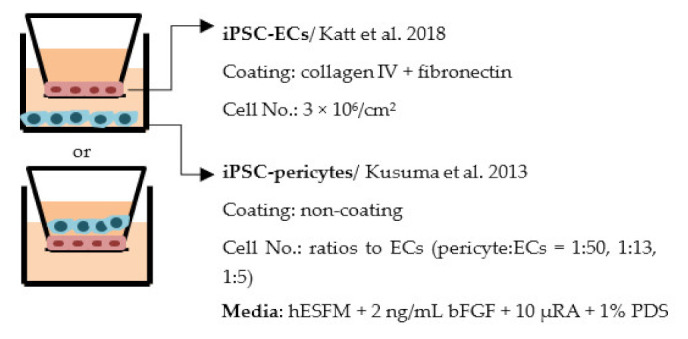	**TEER (Ω × cm^2^)** Monoculture: 3510 Co-culture (non-contact): 3690 (1:50), 3600 (1:13), 3690 (1:5) Co-culture (contact, in gel): 2410 (1:13)
Faal et al. 2019 [209]	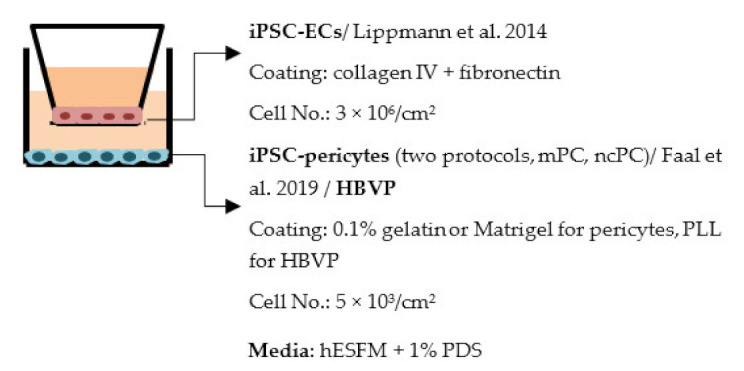	**TEER** Exact values not told, pericytes increased TEER values in co-culture.
Praça et al. 2019 [195]	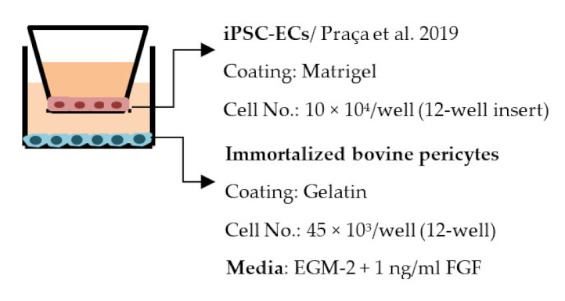	**TEER (Ω × cm^2^)** Mono- and co-culture: ≈60, exact values not told **Permeability, LY (Pe × 10^−3^ cm/min)** ≈0.5, Slight decrease in co-culture compared to optimized monoculture
Blanchard et al. 2020 [107]	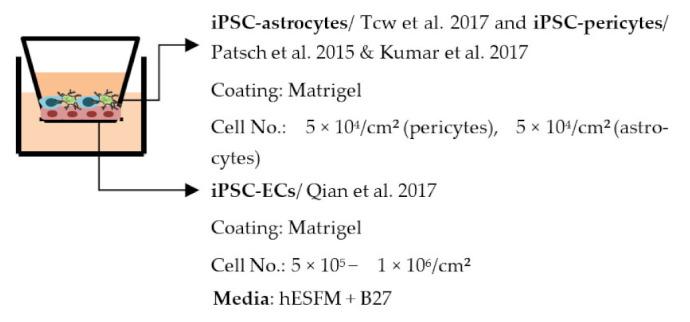	**Average TEER (Ω × cm^2^)** Monoculture: ≈5900 iBBB: ≈8300 **Permeability, Dextran** Values not told, decreased permeability in co-culture **Other** ↑ P-gp in co-culture
Nishihara et al. 2020 [198]	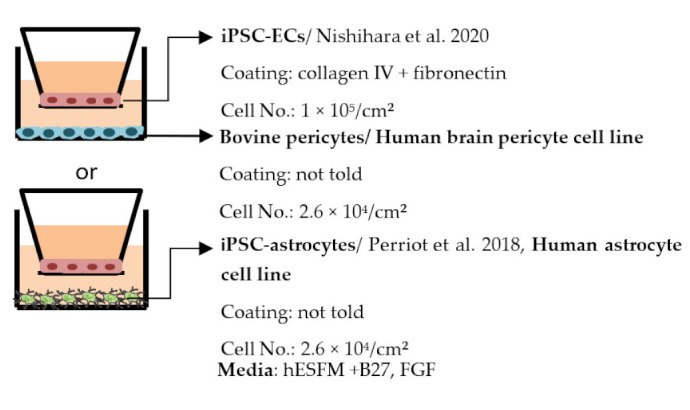	**TEER (Ω × cm^2^)** Monoculture: above 60 Co-culture: similar to monoculture **Permeability, NaF (Pe × 10^−3^ cm/min)** Monoculture: below 0.32 Co-culture: similar to monoculture **Other** more elongated morphology of ECs in co-culture compared to monoculture
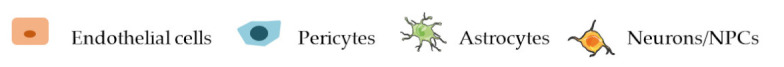		

Abbreviations: **BBB**—Blood–brain barrier; **EC**—Endothelial cells; **ESC**—Embryonic stem cell; **FBS**—Fetal bovine serum; **FGF**—Fibroblast growth factor; **HBVP**—Human brain vascular pericytes; **hESFM**—Human endothelial serum free media; **iBBB**—iPSC-derived BBB; **LY**—Lucifer yellow; **NaF**—Sodium fluoride; **NPC**—Neural progenitor cell; **NSC**—Neural stem cell; **PDS**—platelet-poor plasma-derived serum; **PLL**—Poly-L-Lysine; **RA**—Retinoic acid; **TEER**—Transendothelial electrical resistance; **VEGF**—Vascular endothelial growth factor; **ABAM**—Antibiotic–antimycotic. * Describing co-culture conditions, unless otherwise mentioned; ** Only results from healthy lines included.

**Table 2:** Blood–brain barrier in vitro models (organ-on-chip, hydrogel model, and vascularized brain organoids).

Citation	Experiment Conditions *	Main Readouts **
**Organ on chip**		
Wang et al. 2017 [236]	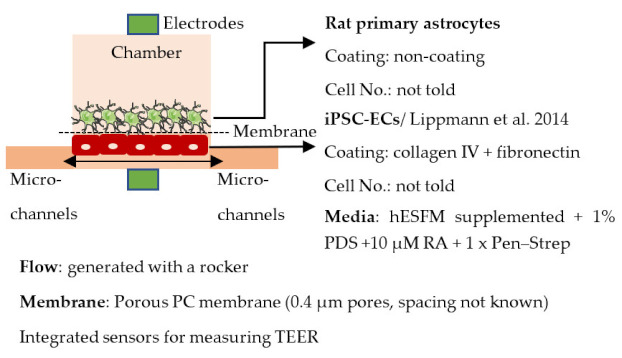	**Max TEER (Ω× cm^2^)** Monoculture: 368 Co-culture: 4399 **Permeability, Dextrans (Papp× 10^−8^ cm/s)** Co-culture: 8.43 (4 kDa), 2.18 (20 kDa) and 0.982 (70 kDa)
Vatine et al. 2019 [237]	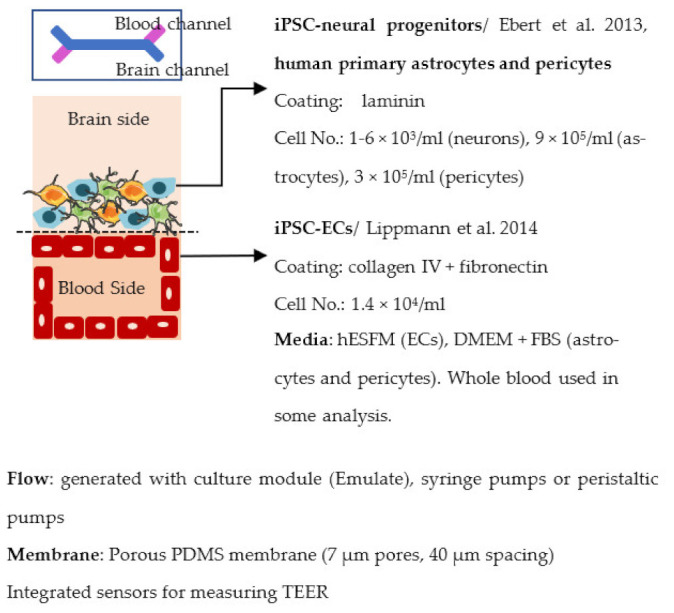	**Max TEER (Ω × cm^2^)** Co-culture (+neural): 1500 **Permeability, Dextran (Papp × 10^−7^ cm/s)** Monoculture: ~3 (exact values not told) Co-cultures (+astrocyte, pericytes/ + neural): ~1 (exact values not told) **Other** ↓ ZO1 expression and ↓ permeability to Dextran after cytokine exposure (+astrocytes, pericytes) Spontaneous neuronal activity (+neural)
Park et al. 2019 [238]	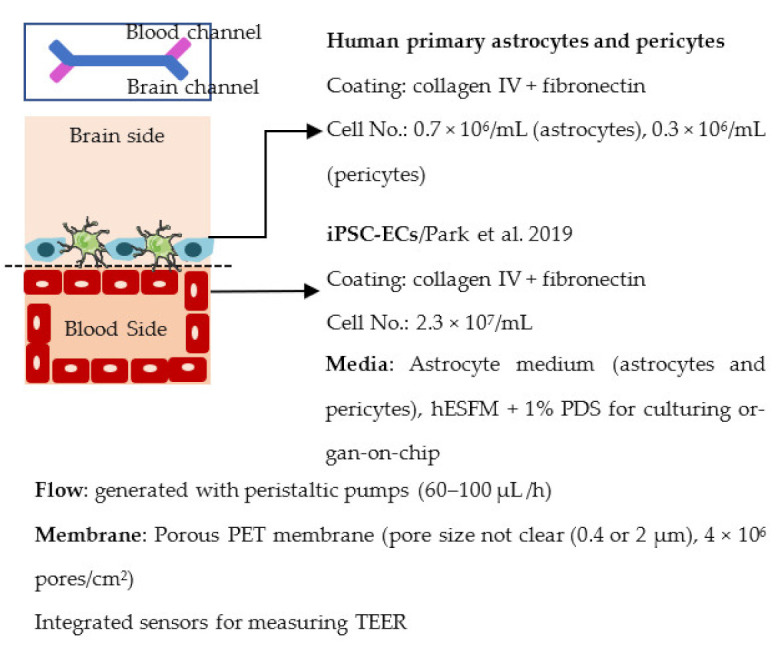	**Impedance (Ω)** ~25 000 **Permeability, Dextran (Papp × 10^−8^ cm/s)** 8.9 (3 kDa), 1.1 (10 kDa) and 0.24 (70 kDa) **Other** Expression and functionality of efflux transporters
Motallebnejad et al. 2019 [239]	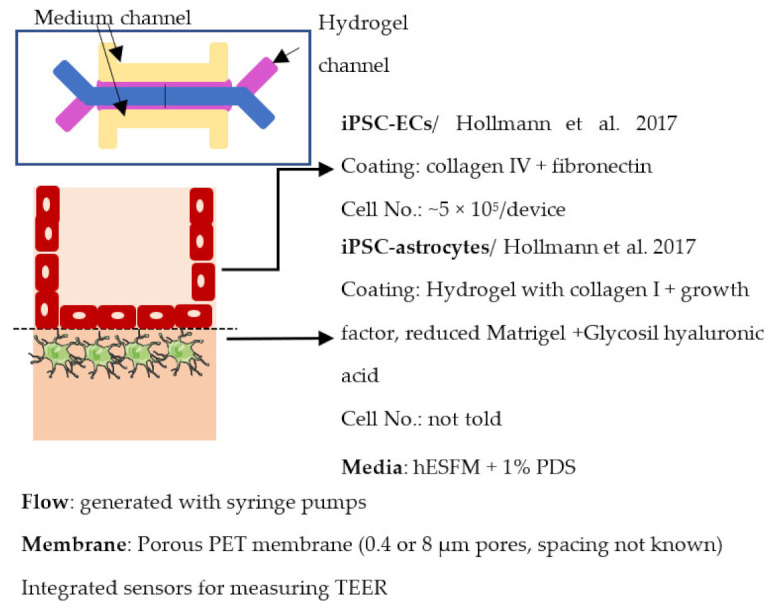	**Max TEER (Ω × cm^2^)** 1590 (0.4 µm membrane), 1369 (8.0 µm membrane) Co-culture increased TEER values **Permeability, NaF (× 10^−6^)** Below 1 **Other** Efflux transporter activity, decreased TEER after TGF-β1 exposure
Pediaditakis et al. 2020 preprint [240]	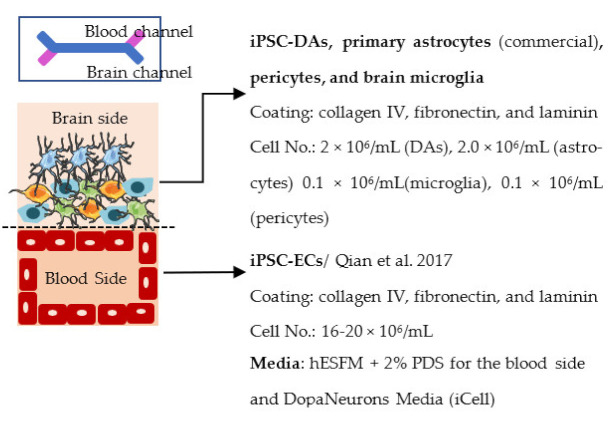	**Permeability (Papp × 10^−6^ cm/s)** range 1–3 (3 kDa), 4–6 (LY) **Other** RNAseq (brain side): more mature phenotype in the chip compared to conventional cell culture, chip recapitulated gene expression profile of primary tissue
**Hydrogels/3D vessels**		
Campisi et al. 2018 [243]	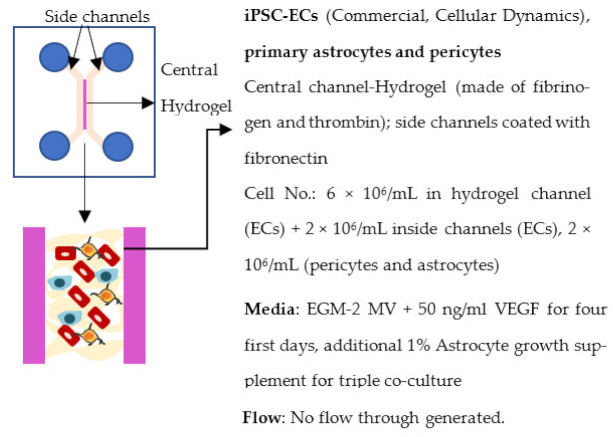	**Permeability coefficient, Dextran (× 10^−7^ cm/s)** Monoculture: 6.6 (40 kDa), 12 (10 kDa) Co-culture (+pericytes): 2.5 (40 kDa), 4.8 (10 kDa) Triple co-culture (+pericytes, astrocytes): 0.89 (40 kDa), 2.2 (10 kDa) **Other** Complex and branched vascular network, ↑ ZO-1, claudin-5 and occludin expression in triple co-culture, ↑ gene expression of several transporters in triple co-culture
Faley et al. 2019 [244]	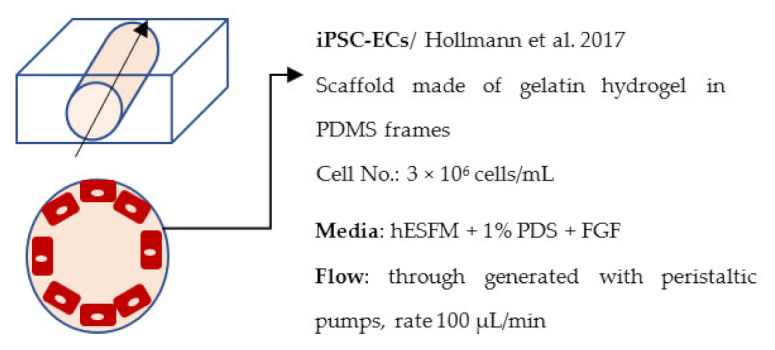	**Permeability, Dextran 3 kDa (× 10^−7^ cm/s)** Day 1: 1,2 (static), 1,9 (perfused) Day 7: 4.6 (static), 1.4 (perfused) Day 14: 11.7 (static), 0.23 (perfused)
Blanchard et al. 2020 [107]	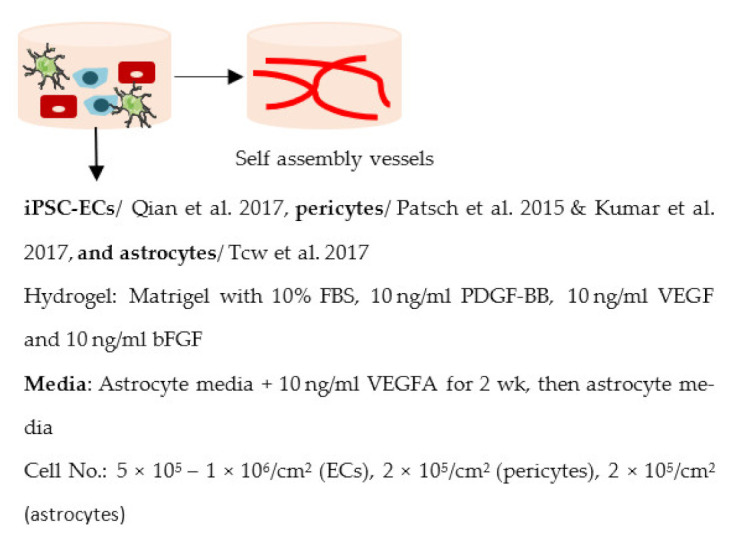	**Other** Capillary structure ↑ CLDN5, JAMA, PGP, LRP1, RAGE and GLUT1 expression in co-culture
**Vascularized organoids**		
Pham et al. 2018 [245]	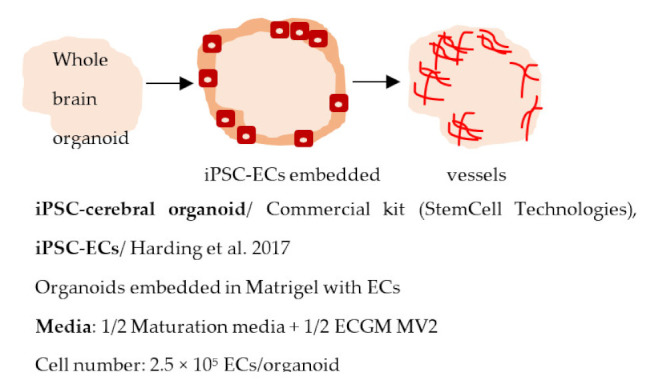	**Other** Tubular structures in organoids, positive for CD31
Cakir et al. 2019 [246]	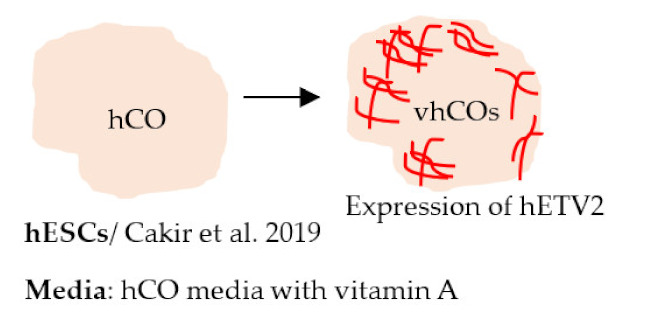	**TEER (Ω × cm^2^)** Day 30: 186 (vhCO), 135 (hCO) Day 70: 351 (vhCO), 71 (hCO) **Other** Presence of vascular tubes positive for CD31, expression of BBB markers (ZO-1, occludin) Presence of astrocytes and pericytes
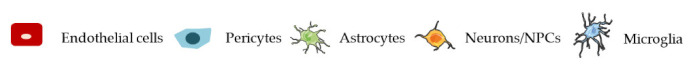		

Abbreviations: **ECs**—Endothelial cells; **ESC**—Embryonic stem cell; **FBS**—Fetal bovine serum; **FGF**—Fibroblast growth factor; **hCO**—Human cortical organoids; **hESFM**—Human endothelial serum free media; **hETV2**—Human ETS variant 2; **LY**—Lucifer yellow; **NaF**—Sodium fluoride; **NPC**—Neural progenitor cell; **PDGF**—Platelet derived growth factor; **PDMS**—Polydimethylsiloxane; **PDS**—Platelet-poor plasma-derived serum; **PET**—Polyethyl terephthalate; **TEER**—Transendothelial electrical resistance; **VEGF**—Vascular endothelial growth factor; **VEGFA**—Vascular endothelial growth factor A; **vhCO**—Vascularized hCO; **Pen-Strep**—Penicillin–streptomycin; **PC**—Polycarbonate; * Describing co-culture conditions, unless otherwise mentioned; ** Only results from healthy lines included.
